# Selective Knockout of the *Vesicular Monoamine Transporter 2* (*Vmat2*) Gene in Calbindin2/Calretinin-Positive Neurons Results in Profound Changes in Behavior and Response to Drugs of Abuse

**DOI:** 10.3389/fnbeh.2020.578443

**Published:** 2020-11-09

**Authors:** Niclas König, Zisis Bimpisidis, Sylvie Dumas, Åsa Wallén-Mackenzie

**Affiliations:** ^1^Unit of Comparative Physiology, Department of Organismal Biology, Uppsala University, Uppsala, Sweden; ^2^Oramacell, Paris, France

**Keywords:** amphetamine, dopamine, locus coeruleus, raphe nuclei, serotonin, substantia nigra, ventral tegmental area, vesicular monoamine transporter (VMAT2)

## Abstract

The vesicular monoamine transporter 2 (VMAT2) has a range of functions in the central nervous system, from sequestering toxins to providing conditions for the quantal release of monoaminergic neurotransmitters. Monoamine signaling regulates diverse functions from arousal to mood, movement, and motivation, and dysregulation of VMAT2 function is implicated in various neuropsychiatric diseases. While all monoamine-releasing neurons express the *Vmat2* gene, only a subset is positive for the calcium-binding protein Calbindin 2 (Calb2; aka Calretinin, 29 kDa Calbindin). We recently showed that about half of the dopamine neurons in the mouse midbrain are positive for Calb2 and that Calb2 is an early developmental marker of midbrain dopamine cells. Calb2-positive neurons have also been identified in other monoaminergic areas, yet the role of Calb2-positive monoaminergic neurons is poorly understood. To selectively address the impact of Calb2-positive monoaminergic neurons in behavioral regulation, we took advantage of the Cre-LoxP system to create a new conditional knockout (cKO) mouse line in which *Vmat2* expression is deleted selectively in Calb2-Cre-positive neurons. In this *Vmat2^lox/lox;Calb2−Cre^* cKO mouse line, gene targeting of *Vmat2* was observed in several distinct monoaminergic areas. By comparing control and cKO mice in a series of behavioral tests, specific dissimilarities were identified. In particular, cKO mice were smaller than control mice and showed heightened sensitivity to the stereotypy-inducing effects of amphetamine and slight reductions in preference toward sucrose and ethanol, as well as a blunted response in the elevated plus maze test. These data uncover new knowledge about the role of genetically defined subtypes of neurons in the brain’s monoaminergic systems.

## Introduction

The monoamine systems of the brain are crucial for normal brain function and their dysfunction is highly correlated with neuropsychiatric and neurological disorders (Ng et al., 2015). Monoamine neurotransmitters, defined by the presence of a single amino group, include the catecholamines—dopamine, noradrenaline, and adrenaline—as well as serotonin and histamine. Though their syntheses differ, these systems share the protein responsible for packaging the neurotransmitter into synaptic vesicles, the vesicular monoamine transporter 2 (VMAT2). A member of the solute carrier (SLC) superfamily of transporter proteins, it is encoded by the *Vmat2* gene, also known as *Slc18a2*.

Several of the brain’s main monoamine systems are located in the midbrain and medulla oblongata, including the midbrain dopamine system comprising the ventral tegmental area (VTA), substantia nigra pars compacta (SNc) and the retrorubral field (RRF), the dorsal raphe (DR) consisting of both dopaminergic and serotonergic neurons, and the locus coeruleus (LC) which is the main brain center for noradrenergic neurons. Common for all these systems is their discrete localization in distinct nuclei, as opposed to glutamatergic and GABAergic neurons that are present throughout the brain. In contrast to the defined presence of cell bodies within brain nuclei, the projections of monoamine neurons are vast and reach large parts of the brain. Through their intricate interactions with many brain areas, monoaminergic neurons exert a strong impact on physiology and behavior. For example, monoamine signaling is associated with motor regulation (Schultz et al., 1989), arousal (Haas et al., 2008; Sara and Bouret, 2012), emotional behaviors (Cools et al., 2008; Likhtik and Johansen, 2019), learning (Keiflin and Janak, 2015) and motivation (Fields et al., 2007; Cools, 2008; Salamone and Correa, 2012). Following their important roles, compromised monoaminergic function is linked to several neurological and neuropsychiatric diseases such as Parkinson’s Disease, substance use disorder, depression, and schizophrenia, as well as brain dopamine-serotonin vesicular transport disease (Christiansen et al., 2007; Gutiérrez et al., 2007; Rilstone et al., 2013; Padmakumar et al., 2019). By removing monoamines from the cytosol, parallel to ensuring vesicular packaging of monoamines, VMAT2 also protects neurons from oxidative stress-related damage (Guillot and Miller, 2009; Lohr et al., 2016). Further, drugs such as reserpine and amphetamine that affect monoamine packaging in synaptic vesicles by acting directly on VMAT2 have profound acute and prolonged effects on behavior by influence on both motor and cognitive functions (Schuldiner et al., 1995; Sulzer et al., 2005).

Experimental studies in transgenic mice exploring the role of VMAT2 have observed various deficits following perturbation of its function, including behavioral changes related to locomotion, anxiety, feeding, and response to drugs. These studies have targeted VMAT2 genetically either by systemic knockout/knockdown strategies (VMAT2 heterozygotes or complete knockouts; Fon et al., 1997; Takahashi et al., 1997; Wang et al., 1997; Mooslehner et al., 2001; Fukui et al., 2007), or by the use of conditional knockout (cKO) strategies based on neurotransmitter phenotype (defined by uptake transporters or synthesis enzymes; Narboux-Nême et al., 2011, 2013; Ohara et al., 2013; Isingrini et al., 2016a,b).

Recently, the selective expression of receptors or transcription factors have been used to direct selective targeting of the *Vmat2* gene (Xu et al., 2017; Bimpisidis et al., 2019). Limiting the intervention to selected VMAT2-positive neuronal populations, leaving the remainder of monoamine signaling cells with unaltered VMAT2 function, allows for probing of the functional role of specific neurons and their circuits. The impact of these more selective alterations can in turn be used to study discrete behavioral phenotypes related to monoamine dysfunction such as stress, depression, and movement disorders.

The calcium-binding protein Calbindin 2 (Calb2, also known as Calretinin and 29 kDa Calbindin) is present in a range of structures in the brain—including, but not limited to, the cortex, hypothalamus, midbrain, pons, and medulla—and is implicated in several functions including developmental processes and neuroprotection (Barinka and Druga, 2010; Schwaller, 2014; Fairless et al., 2019). In the midbrain dopamine system of rodents and primates, some, but far from all, dopamine neurons of the VTA and SNc are positive for *Calb2* gene expression (Rogers, 1992; Isaacs and Jacobowitz, 1994; Mouatt-Prigent et al., 1994; Fortin and Parent, 1996; Liang et al., 1996; McRitchie et al., 1996; Nemoto et al., 1999; Poulin et al., 2014; Viereckel et al., 2016; Mongia et al., 2019).

Calb2-positive dopamine neurons thus form a subtype of dopamine neurons, joined by their expression of the *Calb2* gene. Following up on data obtained in a microarray analysis of the mouse midbrain, by comparing expression in the VTA with the SNc using histological methods we have previously shown that Calb2 mRNA is prominent in both the VTA and the SNc, with the strongest signals detected in lateral VTA, rostral linear nucleus (RLi) and SNc, and somewhat weaker in the medially positioned interfascicular nucleus (IF; Viereckel et al., 2016). To further address this Calb2-population, we recently used fluorescent *in situ* hybridization to allow co-detection and could show that about 50% of the dopamine neurons in the mouse VTA are positive for Calb2 mRNA. Calb2-positive dopamine neurons were identified throughout all VTA subareas: the IF, parainterfascicular nucleus (PIF), paranigral nucleus (PN), parabrachial pigmented nucleus (PBP), and rostral VTA (VTAR; Bimpisidis et al., 2019). This finding is coherent with previous immunohistochemical observations (Liang et al., 1996). Calb2 mRNA was also detected in scattered dopamine neurons throughout the SNc. In addition to dopamine neurons, Calb2 mRNA was identified in glutamate and GABA neurons within the VTA and SNc. Calb2-positive neurons thus show a heterogeneous neurotransmitter phenotype in the VTA and SNc (Bimpisidis et al., 2019). By implementing a protocol for optogenetic stimulation in the VTA coupled with behavioral testing in a place preference paradigm (Bimpisidis et al., 2020), no significant response was obtained when Calb2-Cre mice were tested as opposed to when stimulating other subtypes of VTA dopamine neurons (Bimpisidis et al., 2019). This finding was surprising, given the amount of Calb2-positive neurons in the VTA area, but may be explained by a low amount of extra-VTA projections, tentatively suggesting that Calb2-positive VTA neurons primarily interact with other VTA neurons (Bimpisidis et al., 2019). This was the first study probing the functional role of Calb2 cells in the VTA which highlighted the need for specific and varied experimental approaches to address the functional role of Calb2-positive neuronal populations.

Further following up on the identification of Calb2-positive neurons in the ventral midbrain of the adult mouse, we recently addressed the embryonal development of this same brain area. We found that Calb2 mRNA can be detected already at embryonal day 14.5 (E14.5), shortly after the neurons acquire their dopamine phenotype (Dumas and Wallén-Mackenzie, 2019). Calb2 was not detected at E11.5, however, suggesting that the onset of Calb2 expression is between E11.5 and E14.5. Beyond the midbrain dopamine system, Calb2 has also been observed in other monoamine areas, including several raphe nuclei, and in hypothalamic dopaminergic cells; in contrast, Calb2 has been demonstrated as absent in the noradrenergic LC (Arai et al., 1991; Résibois and Rogers, 1992). However, little is known about the role of Calb2-positive monoaminergic neuronal populations in behavioral regulation.

In this study, we aimed to anatomically and functionally probe the Calb2 subtype of monoaminergic neurons. We implemented histological and gene-targeting approaches followed through with behavioral assessments and pharmacological challenges. Upon confirmed deletion of *Vmat2* gene expression selectively in Calb2-Cre-positive neurons, behavioral analysis of this new *Vmat2^lox/lox;Calb2−Cre^* line of *Vmat2* cKO mice revealed significantly altered behaviors in terms of locomotion, anxiety, and responses to sucrose, ethanol, and amphetamine. The results demonstrate that the Calb2-positive subtype of monoaminergic neurons is crucial for normal behavior.

## Materials and Methods

### Mice

**Figure 1:**
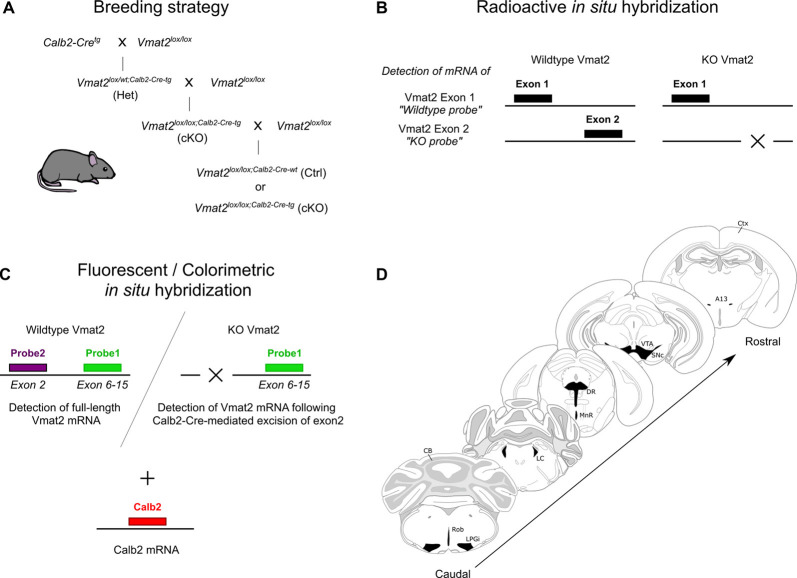
Illustrations of the experimental approach. Schematic of breeding strategy to produce Calb2-directed VMAT2 conditional knockouts (cKO) using a Cre-LoxP system **(A)**. Radioisotope-labeled *in situ* hybridization **(B)**, and three-probe fluorescent/colorimetric *in situ* hybridization **(C)** method to verify *Vmat2* recombination and Calb2 co-localization. The span of sections analyzed with illustrated monoaminergic areas **(D)**. CB, cerebellum; Ctx, cortex; DR, dorsal raphe; LC, locus coeruleus; LPGi, lateral paragigantocellular nucleus; MnR, median raphe; Rob, raphe obscurus; SNc, substantia nigra compacta; VTA, ventral tegmental area.

All experiments were conducted according to Swedish (Animal Welfare Act SFS 1998:56) and European Union legislation (Convention ETS 123 and Directive 2010/63/EU) and following Uppsala Ethical Committee for Laboratory Animal Research. The mice were group-housed (2–5 mice per cage) in a temperature- and humidity-controlled animal husbandry room, on a 12:12 light:dark cycle (lights on at 06:00 AM), and provided with food and water *ad libitum*. Heterozygous Calb2-Cre transgenic mice (The Jackson Laboratory, Calb2^tm1(cre)Zjh^/J) were bred with *Vmat2^lox/lox^* mice, which have exon 2 of the *Vmat2* gene flanked by LoxP sites (Narboux-Nême et al., 2011). This strategy allowed for generation of (cKO; *Vmat2^lox/lox;Calb2−Cre−tg/wt^*) and Cre-negative controls (Ctrl; *Vmat2^lox/lox;Calb2−Cre−wt/wt^*; Figure 1A). Genotyping was performed by PCR with the following primer sequences: Calb2-Cre 5′-ACG AGT GAT GAG GTT CGC AAG A-3′; 5′-ACC GAC GAT GAA GCA TGT TTA G-3′; Vmat2lox 5′-GAC TCA GGG CAG CAC AAA TCT CC-3′; 5′-GAA ACA TGA AGG ACA ACT GGG ACC C-3′. Control and cKO adult mice (>8 weeks) of both sexes were used for the experiments.

### Histological Analysis

#### *In Situ* Hybridization (ISH) and Fluorescent *In Situ* Hybridization (FISH)

Brains were dissected from deeply anesthetized cKO and control mice at 17 days of age, and snap-frozen in isopentane (2-Methylbutane) on dry ice. Sections were cut on a cryostat and slides were prepared for *in situ* hybridization.

Experiments were performed as previously reported (Bimpisidis et al., 2019). Briefly, radioactive oligoprobes (for ISH; Figure 1B) and riboprobes (for FISH) or combined FISH/brightfield ISH; Figure 1C) were used. The following oligoprobe sequences were used: Calb2: NM_00786.1; bases 33–61, 946–979; and 1299–1332. Th: NM_009377.1; bases 774–807, 272–305, 1621–1655. Vmat2exon1 (wildtype probe): NM_172523.3; bases18–51 and 83–116. Vmat2exon2 (KO probe): NM_172523.3; bases 201–237 and 240–276. The following riboprobe sequences were used: Calb2: NM_007586.1; bases 80–793. Vmat2 Probe1: Vmat2: NM_0130331.1 (rat); bases 701–1439 (corresponds to exon 6–15 of mouse sequence NM_172523.3). Vmat2 Probe2: NM_172523.3; bases142–274 i.e., the whole exon 2. The latter riboprobes targeting different exons of the Vmat2 mRNA termed Vmat2 Probe1 and Vmat2 Probe2 were used for detection of wildtype and recombined Vmat2 DNA as described below.

#### Validation of Recombination of Floxed Vmat2 Exon 2 Driven by the Calb2-Cre Transgene

A three-probe *in situ* hybridization approach was employed to visualize monoaminergic Calb2-positive cells (Figure 1C). In addition to labeling with a fluorescent probe for Calb2 mRNA, two probes were used to visualize Vmat2 mRNA as previously reported (Bimpisidis et al., 2019). Vmat2 mRNA derived from exon 6–15 was detected by Probe1 (green fluorescent), whereas Probe2 (purple/black) detected mRNA from exon 2. In the case of *Vmat2* wildtype cells, both probes bind to Vmat2 mRNA giving a combination of green and purple/black labeling. In those cells in cKO mice in which recombination has taken place, only Probe1 (green) can bind as exon 2 has been excised. Thus, knockout (KO) cells with abrogated Vmat2 are visualized by a green signal and a lack of purple/black signal.

#### Quantification of Vmat2 Knockout Cells

Serial sections from two mice of each genotype were analyzed for the occurrence of the recombined *Vmat2* allele (binding of only Probe1, *Vmat2* KO cells) *vs.* control (binding of both Probe1 and Probe2, *Vmat2* wildtype cells). Manual counting of cells positive for Probe1 *vs.* Probe1 + Probe2 was performed with Probe1 used as a reference for anatomical boundaries and outline of distinct cell soma.

#### Immunohistochemistry

Deeply anesthetized adult cKO and control mice were transcardially perfused with room-temperature PBS followed by ice-cold 4% formaldehyde. Brains were dissected and post-fixed overnight, transferred to PBS, and sectioned at 60 μm using a vibratome. Free-floating sections were processed for immunohistochemistry according to standard protocols. After a series of washes with PBS and PBS containing 0.1% Triton-X, they were incubated for 1 h in an appropriate blocking solution (5% serum in 0.1% PBS-T) at room temperature (RT). Incubation of primary antibodies diluted in 0.1% PBS-T with 5% serum took place overnight at 4°C [rabbit anti-TH 1:1,000, Millipore, #MAB318; rabbit anti-TPH2 1:1,000 Novus Biologicals #NB100–74555]. Sections were subsequently washed in 0.1% PBS-T and incubated with a biotinylated goat anti-rabbit antibody (ABC kit; Vector laboratories #PK-4001) in 0.1% PBS-T for 1.5 h at RT. After subsequent washing steps, DAB (Vector Laboratories) was used as a chromogen. The sections were mounted on glass slides, incubated in cresyl violet, dehydrated in a series of ascending ethanol solutions, and coverslipped using DPX (Sigma–Aldrich, 06522). Images were captured using a NanoZoomer S60 scanner and processed using the Ndp2.view software (Hamamatsu) or ImageJ.

### Behavioral Analysis

#### Baseline Locomotion

Mice were placed in polycarbonate boxes (Makrolon), containing 1.5-cm bedding, covered with a transparent, perforated plexiglass lid (cKO *n* = 21, control *n* = 17). Spontaneous horizontal activity and habituation to a novel environment were recorded for 60 min by the EthovisionXT (Noldus) tracking software. Data are expressed as distance moved during the recording period.

#### Sucrose Preference Test

Mice were housed individually and were presented with two drinking bottles (cKO *n* = 9, control *n* = 6). After 48 h of habituation to the experimental setup, the mice were presented with one bottle containing tap water and one sucrose solution (1, 3, and 10%). Each bottle was weighed and replaced every 24 h. Each sucrose concentration was tested twice (i.e., two consecutive days) and the position of the bottles was alternated to exclude side biases. Preference for the sucrose bottle was calculated as the percentage of volume consumed from the sucrose bottle compared with total liquid consumption (from both sucrose and water bottle).

#### Ethanol Preference Test

The experimental setup was similar to the one described for the sucrose preference test (cKO *n* = 16, control *n* = 16). After habituation to two water bottles, the mice were introduced to one bottle of tap water and one containing a solution of increasing concentration of ethanol (3, 6, and 10%). The bottles were weighed and changed every 24 h, with alternating positions, and each concentration was tested four times (i.e., four consecutive days of each concentration). Preference for the ethanol bottle was calculated as the percentage of volume consumed from the ethanol bottle compared with total liquid consumption (from both ethanol and water bottle).

#### Elevated Plus Maze

Animals were placed on an elevated plus maze with two open and two closed arms for 5 min without prior habituation to the apparatus (cKO *n* = 21, control *n* = 22). Mice were positioned close to and facing away from the center on one of the open arms. During the recording time, EthovisionXT (Noldus) tracking software was used to monitor their behavior, including time spent in each compartment and transitions between them.

#### Rotarod

Motor coordination assessment took place on a Rotarod apparatus (Panlab) under an incremental fixed speed schedule and a session with accelerating rotation speed. On consecutive days, mice were trained at rotation speeds of 4, 8, and 16 RPM (cKO *n* = 17, control *n* = 16). Latency to fall at each speed was noted during three trials, separated by 30 min, with three attempts per trial. A trial was ended when all three attempts were made, or after a maximum of 120 s was achieved. For the session with accelerating rotation speed, latency to fall was noted during three trials, separated by 30 min, with a rotation speed increasing from 4 to 40 RPM over 5 min (cKO *n* = 8, control *n* = 8). Statistical analysis was performed on the maximum value achieved during each trial.

#### Amphetamine Sensitization

The behavior of the mice upon injections of amphetamine or saline was monitored in boxes as described above for baseline locomotion. Mice received intraperitoneal injections of saline (day 1, “Saline”) followed by 3 mg/kg amphetamine (days 2–5, “Amph 1–4”), and a final injection of the same dose of amphetamine on day 17 (“Challenge”; cKO *n* = 13, control *n* = 10). Horizontal activity was recorded 30 min before and 90 min following injection, using EthovisionXT (Noldus).

### Statistical Analysis

Appropriate statistical tests were performed in GraphPad PRISM version 8. For comparisons of two factors (such as genotype and time), repeated measure two-way ANOVA was used, with *post hoc* tests for testing within factor means. Simple averages between groups were tested using Student’s *t*-test or Mann–Whitney test.

## Results

The presence of VMAT2 is restricted to monoamine-releasing cells. To delete the ability for monoamine vesicular packaging selectively in Calb2-positive cells, a Cre-LoxP strategy was utilized in which expression of Cre recombinase is driven by promotor sequences for the *Calb2* gene (Figure 1A). By crossing the Calb2-Cre-line with a floxed allele of *Vmat2*, in which exon 2 of the *Vmat2* gene is surrounded by LoxP sites (Narboux-Nême et al., 2011), we generated a new cKO mouse line in which the floxed sequence containing exon 2 of the *Vmat2* gene is excised specifically in Calb2-Cre cells. Throughout the experiments, PCR-verified cKO mice positive for Calb2-Cre and homozygous for the floxed allele (*Vmat2^lox/lox;Calb2−Cre−tg^*; cKO) were compared with littermates homozygous for the floxed allele but negative for Calb2-Cre (*Vmat2^lox/lox;Calb2−Cre−wt^*; Ctrl).

### Histological Evaluation of Calb2-Cre-Driven Targeting of the Vmat2 Allele

Using two different *in situ* hybridization approaches (Figures 1B,C), we confirmed the expression of *Vmat2* and its targeted deletion. Multiple brain sections in series were analyzed encompassing major monoaminergic systems in the fore-, mid-, and hindbrain, including the hypothalamus, VTA, SNc, RRF/caudal linear nucleus (RRF/CLi), LC, and raphe nuclei (Figure 1D).

**Figure 2:**
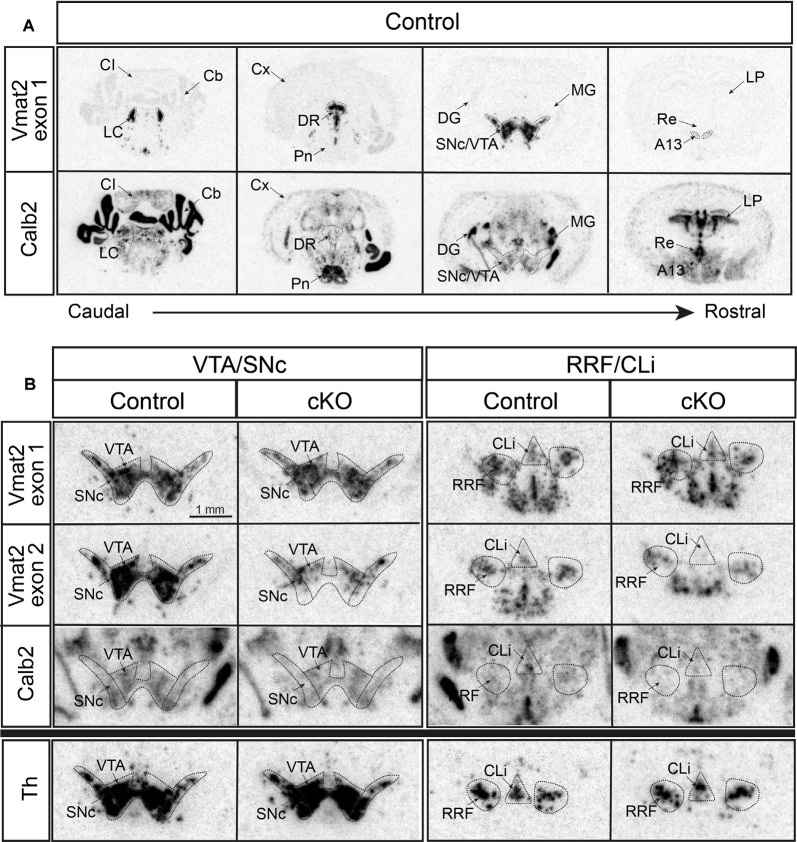
Histological validation of cKO of VMAT2 in Calb2-driven Cre-producing cells by radioisotope-labeled *in situ* hybridization. Detection of Vmat2 exon 1 (using “wildtype” probe) and Calb2 mRNA in various monoaminergic areas of the brain in control mice **(A)**. Detection of Vmat2 exon 1 (using “wildtype” probe), Vmat2 exon 2 (using “KO” probe), Calb2, and Th mRNA in midbrain dopaminergic areas for control and cKO mice **(B)**. Cb, cerebellum; CI, inferior colliculus; Cli, caudal linear nucleus; Cx, cortex; DG, dentate gyrus; DR, dorsal raphe; IF, interfascicular nucleus; LC, locus coeruleus; LP, lateral posterior thalamic nucleus; MG, medial geniculate nucleus; PBP, parabrachial nucleus; PIF, parainterfascicular nucleus; Pn, pontine nuclei; Re, reuniens nucleus; RLi, rostral linear nucleus; RRF, retrorubral field; SNc, substantia nigra compacta; Th, tyrosine hydroxylase; VTA, ventral tegmental area.

First, radioisotope-labeled *in situ* hybridization was performed to grossly detect mRNAs. Calb2 was confirmed in the VTA and SNc, and also identified with a vast presence in subcortical areas, midbrain, medulla, and cerebellum (Figure 2A). Two different *Vmat2* probes were used to address Vmat2 expression and allow detection of the Vmat2 KO allele: one “wildtype probe” detecting the wildtype *Vmat2* allele (exon 1) and one “KO probe” detecting the *Vmat2* exon2 KO allele (normal in wildtype cells and excised in KO cells; Figure 1B). As expected, in control mice, both the wildtype and KO probes were detected at similar intensity across all analyzed monoaminergic areas (Figures 2A,B, Supplementary Figure 1). In contrast, a weaker signal was observed from the *Vmat2* KO probe in monoaminergic areas of the cKO mice compared to controls. With this method, the lower detection was particularly evident in the VTA and SNc (Figure 2B). Detection levels of Calb2 mRNA were generally similar between genotypes (Supplementary Figure 1). Tyrosine hydroxylase (Th) mRNA was analyzed to address the integrity of catecholaminergic neurons. Similar Th mRNA levels in control and cKO mice were found throughout all catecholaminergic areas including the VTA, SNc, RRF/CLi, and LC (Figure 2B; Supplementary Figure 1).

**Figure 3:**
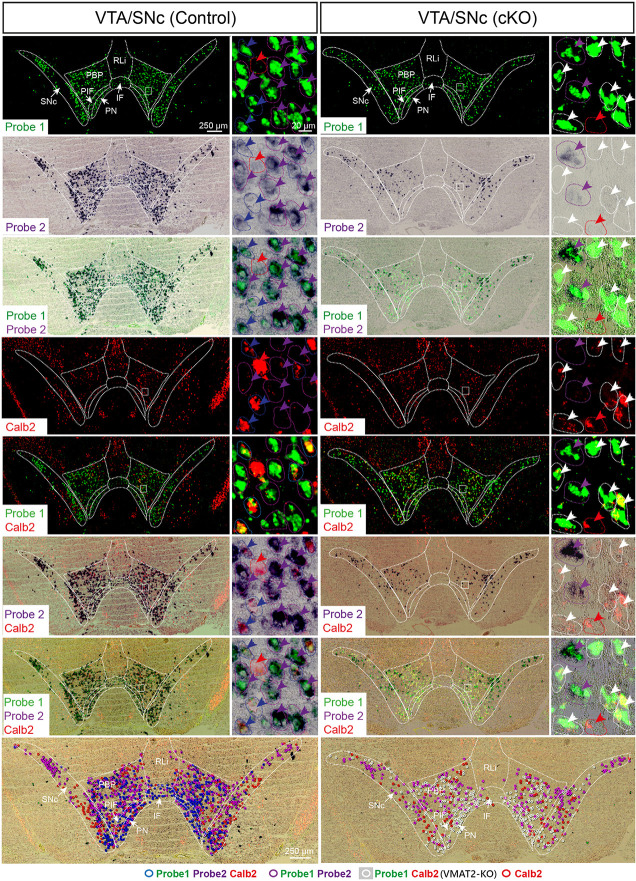
Three-probe *in situ* hybridization in midbrain dopaminergic areas. Wildtype cells are identified by binding of probes to mRNA of exons 6–15 (Probe1; green) as well as exon 2 (Probe2; purple/black), whereas green signal alone identifies knockout (KO) cells in which recombination has led to the removal of exon 2. Additionally, co-localization with Calb2 (red) was studied. Arrow colors correspond to the legend provided at the bottom of the figure, where different combinations of expression patterns are symbolized and superimposed on the image to show their distribution (bottom row). Blue circles symbolize cells with wildtype Vmat mRNA co-localizing with Calb2 mRNA; purple circles symbolize cells with wildtype Vmat2 negative for Calb2; white circles symbolize VMAT2-KO cells; red circles symbolize non-monoaminergic Calb2-positive cells. IF, interfascicular nucleus; PBP, parabrachial nucleus; PIF, parainterfascicular nucleus; PN, paranigral nucleus; RLi, rostral linear nucleus; SNc, substantia nigra compacta; VTA, ventral tegmental area.

**Figure 4:**
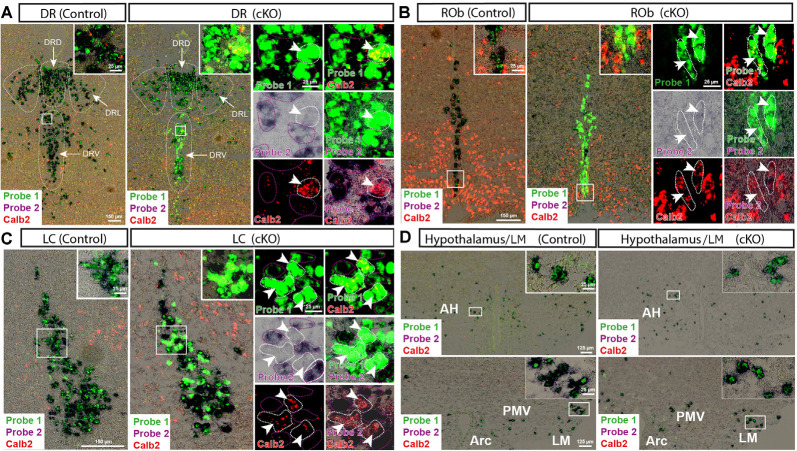
Detection of Vmat2 deletion and Calb2 co-localization in additional areas in the brain. Insets show high magnification of signals from Vmat2 Probe1 (green), Probe2 (purple/black), and Calb2 (red) in the ventral and lateral portions of the dorsal raphe (DRV; DRL; **A**), nucleus raphe obscurus (ROb; **B**), LC **(C)**, and hypothalamic areas **(D)**. White arrows indicate KO cells positive for Vmat2 Probe1 and Calb2, but negative for Vmat2 Probe2. AH, anterior hypothalamus; Arc, arcuate nucleus; DR, dorsal raphe; DRL, lateral portion of dorsal raphe; DRD, dorsal portion of dorsal raphe; LM, lateral mammillary nucleus; PMV, ventral premammillary nucleus.

With the confirmed detection of Vmat2 exon2-deletion in the midbrain VTA and SNc neurons, a more careful analysis was performed to ascertain the histological phenotype of cKO brains. For this purpose, fluorescent *in situ* hybridization (FISH) was implemented to allow the co-detection of several mRNAs. First, we sought to address the presence of Calb2 mRNA to identify areas in which the *Vmat2* gene might be deleted (summarized in Table 1, Figures 3, 4). In the midbrain, in addition to the VTA and SNc (Figure 3, Table 1), Calb2 mRNA was identified in the RLi, RRF and CLi (Figures 2B, 3), and several raphe nuclei, including the dorsal, lateral, and ventral dorsal raphe (DRD, DRL, DRV; Figure 4A), median raphe (MnR), raphe Magnus/raphe interpositus (RMg/RIP), interfascicular nucleus (DRI), and raphe obscurus (Rob; Figure 4B). Caudally, Calb2 mRNA was found in the LC (Figure 4C), the lateral paragigantocellular nucleus (LPGi), and in distinct adrenergic subareas (C1–C3) of the medulla oblongata (Table 1). Further, rostrally, low levels of Calb2 mRNA, or its absence, were detected in the mammillary and premammillary nuclei, arcuate nucleus, and anterior hypothalamus (AH; Table 1, Figure 4D) as well as hypothalamic dopamine neurons of area A13 (Table 1).

**Table 1:** Extent of recombined (knockout) cells and Calb2/Vmat2 distribution in the brain of *VMAT2^lox/lox;Calb2−Cre−tg^* (cKO) mice.

	Region		Vmat2 KO cells	Calb2/Vmat2 coloc	Calb2
Hypothalamus	A13 dopamine cells		0	0
	Anterior hypothalamus (AH)		0	0	
	Arcuate nucleus		+	+	Weak or no signal
Premammillary nucleus	PMV		0	0
Mammillary nucleus	LM		0	0	
Dopaminergic cells (A8–A9–A10)	VTA (A10)	IF/PN/PIF	++/+++	++/+++	
		PBP	+/++	+/++	
	SNc (A9)	Ventral SNc	+++	+++	
		Medial and lateral SNc	+	+	
	RRF (A8)		++/+++	++/+++	
Linear Nuclei	RLi		+	+	
	CLi		++/+++	++/+++	
Raphe nuclei	DR	DRD	0	0	Yes
		DRL	+	+
		DRV	++/+++	++	
	MnR		++/+++	++	
	DRI		++/+++	++/+++	
	RMg/RIP		++/+++	++/+++	
	Rob		+++	++	
Noradrenergic cells	LC	Rostro-ventral part	+	0/+	
		Rostro-dorsal part and caudal	++	+	
Adrenergic cells	LPGi		++/+++	++/+++	
	C1–C2–C3		+	+	

*Symbols express range of percent accordingly: 0, + (0–30%), ++ (30–60%), +++ (60–90%), ++++ (90–100%). Cli, caudal linear nucleus; DRD, dorsal part of dorsal raphe; DRI, interfascicular dorsal raphe; DRL, lateral part of dorsal raphe; DRV, ventral part of dorsal raphe; IF, interfascicular nucleus; LC, locus coeruleus; LM, lateral mammillary nucleus; LPGi, lateral paragigantocellular nucleus; MnR, median raphe; PBP, parabrachial nucleus; PIF, parainterfascicular nucleus; PMV, ventral premammillary nucleus; PN, paranigral nucleus; Rob, raphe obscurus; RLi, rostral linear nucleus; RMg/RIP, raphe magnus/raphe interpositus; RRF, retrorubral field; SNc, substantia nigra compacta; VTA, ventral tegmental area*.

Calb2/Vmat2 co-localization analysis next showed that Calb2 mRNA colocalized with Vmat2 mRNA throughout these areas: VTA, SNc, RRF, RLi, CLi, DR (mostly DRV), MnR, DRI, RMg/RIP, Rob, LC, LPGi, C1–3, and arcuate nucleus. No Calb2/Vmat2 co-localization was seen in hypothalamic nuclei, mammillary, and premammillary nuclei. Also, the dorsal aspect of the DR, DRD, was devoid of Calb2/Vmat2 colocalization. In areas where Calb2 and Vmat2 mRNAs did colocalize, the level varied between areas (Figures 3, 4, and Table 1).

To assess the extent of Calb2-Cre-driven recombination of the floxed *Vmat2* allele, all areas identified as positive for Calb2/Vmat2 in control mice were next analyzed for the presence of Vmat2 KO cells in the cKO mice. Here, to identify the precise location and extent of recombination patterns with high anatomical resolution, we employed a three-probe *in situ* hybridization approach. Labeling of Calb2 mRNA (red) was combined with two different Vmat2 probes to allow the detection of both wildtype and KO Vmat2 mRNA (Figure 1C). Cells with wildtype Vmat2 mRNA in both control and cKO mice were visualized by the binding of both a fluorescent probe (green; Probe1, binding to exon 6–15) and a colorimetric probe [purple/black; Probe2, binding to exon 2 (the floxed exon)]. In contrast, in cells in which Calb2-Cre-mediated recombination of the floxed *Vmat2* exon 2 has taken place, probe 2 fails to bind. Vmat2 KO cells could thereby be detected by their clear green fluorescence due to the binding of only Probe 1. This strategy allowed Vmat2 KO cells to be readily visualized.

Consistent with the observed sites of Calb2/Vmat2 co-localization, Vmat2 KO cells were detected in cKO mice in all brain areas listed above as positive for Calb2/Vmat2 in control mice: VTA, SNc, RRF, RLi, CLi, DR (mostly DRV), MnR, DRI, RMg/RIP, Rob, LC, LPGi, C1–3, as well as the arcuate nucleus (Table 1, Figures 3, 4). The level of recombination (i.e., proportion of Vmat2-positive cells positive for only Probe1) was similar to the level of co-localization in control mice (Table 1, and described further below). In contrast, consistent with their lack of Calb2/Vmat2 co-localization, Vmat2 mRNA in hypothalamic nuclei, mammillary and premammillary nuclei as well as DRD was unaffected and appeared similar in control and cKO mice (Table 1, Figures 4A,D).

Careful analysis showed a high occurrence of Vmat2 deletion in midbrain dopamine cells in cKO mice. Following the results of our previous study (Bimpisidis et al., 2019), approximately half of the VTA/SNc cells were also positive for Calb2 mRNA (Probe1+/Probe2+/Calb2+), and these were positive for the Vmat2 KO mRNA in cKO mice (Probe1+/Probe2−/Calb2+). Rare KO cells did not show detectable levels of Calb2 (Probe1+/Probe2−/Calb2−), a finding which might reflect developmental *Calb2* expression not detected in the adult but which contributed with Calb2-Cre activity. Within the VTA, KO cells were present in all subregions, the IF, PIF, PN, PBP, and VTAR, following the previously described distribution of Calb2 mRNA (Figure 3). Extensive *Vmat2* recombination was detected in the ventral part of the SNc, corresponding to the higher levels of Calb2 mRNA here than in dorsolateral SNc (Figures 2B, 3, also Bimpisidis et al., 2019). Further, a few Vmat2 KO cells were found in the RLi, and a moderate to an extensive amount of KO cells was detected in both the RRF and CLi (Table 1). Moderate to extensive *Vmat2* recombination was observed in the raphe nuclei, including DR (with most in DRV), MnR, DRI, RMg/RIP, Rob (Figure 4, Table 1). The LPGi also contained moderate to extensive proportions of Vmat2 KO cells, and a few knockout cells were observed in the adrenergic cells of the medulla, as well as the arcuate nucleus (Table 1).

Overall, Calb2-Cre-driven targeting of the *Vmat2* gene was confirmed in ample midbrain and hindbrain monoaminergic areas in a pattern that largely follows the endogenous expression of Calb2 mRNA. The patterns and the level of recombination were following the patterns and amount of cells co-expressing the *Calb2* and *Vmat2* genes, thus verifying the efficiency of the cKO strategy.

**Figure 5:**
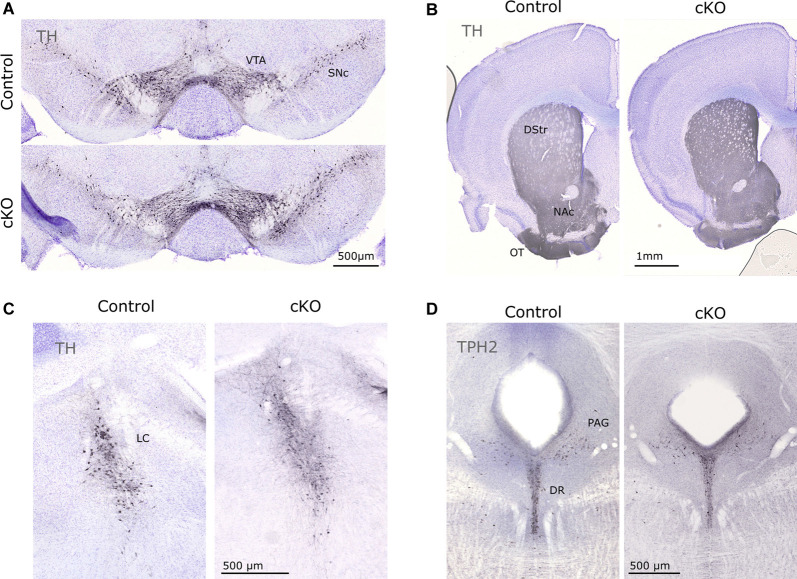
Immunohistochemical evaluation of control and cKO brain tissue. TH was used to visualize cell bodies of dopaminergic cells in the VTA and SNc **(A)** and projections in the striatum **(B)**, as well as catecholaminergic neurons in the LC **(C)**. TPH2 identifies serotonergic cell bodies in the DR **(D)**. DR, dorsal raphe; DStr, dorsal striatum; LC, locus coeruleus; NAc, nucleus accumbens; OT, olfactory tubercle; PAG, periaqueductal gray; TH, tyrosine hydroxylase; TPH2, tryptophan hydroxylase 2.

Finally, immunohistochemistry for TH and TPH2 (as markers for catecholaminergic and serotonergic neurons, respectively) did not reveal any gross anatomical differences in the mesostriatal system, LC, or in the DR (Figure 5). Together with the stable presence of Th and Calb2 mRNA (above), this finding confirmed consistent cytoarchitecture despite the prominent loss of Vmat2 mRNA.

### Changes in Weight and Basal Locomotion, but Not in Motor Coordination

**Figure 6:**
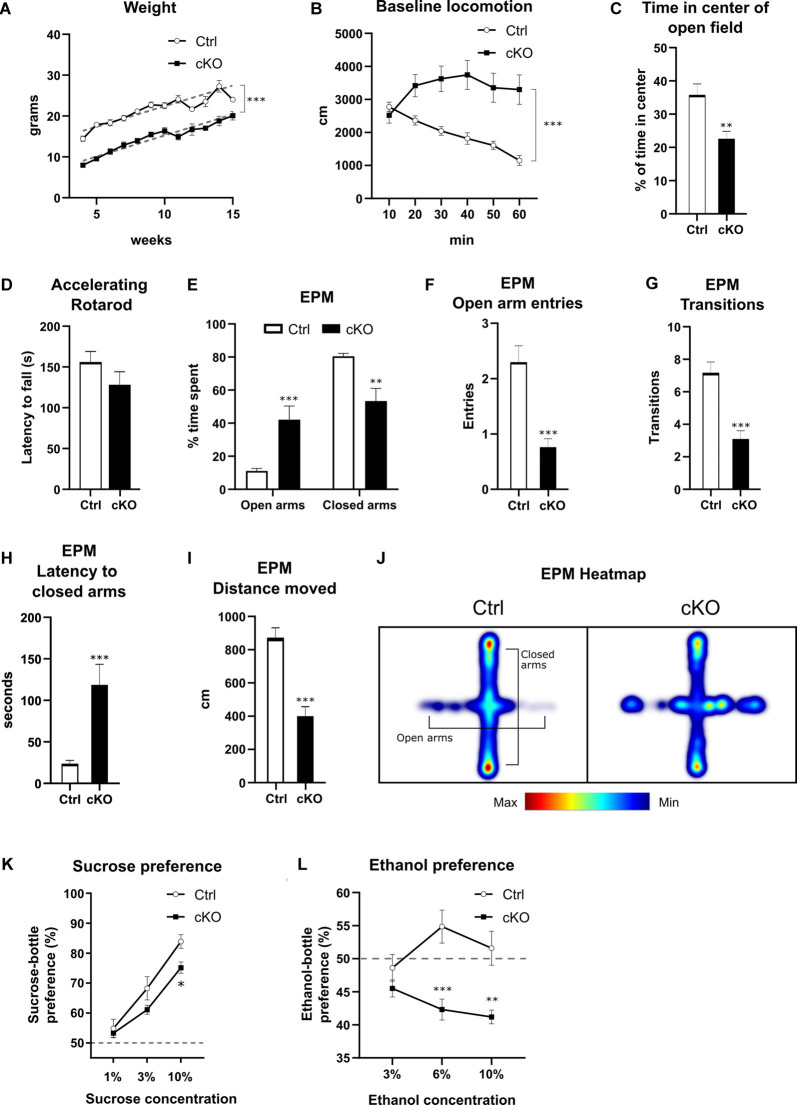
Behavioral characterization of cKO mice and littermate controls in various aspects of development and behavior. Weight curves **(A)** with lines of best fit (gray dashed lines). Horizontal locomotion during 60 min in a novel open field environment **(B)**, analyzed for time spent in the center of the arena **(C)**. Latency to fall from a rotarod at accelerating speeds **(D)**. Various parameters measured in the elevated plus maze **(E–I)**: the proportion of time spent in open *vs.* closed arms **(E)**, number of entries into open arms **(F)**, overall transitions in all compartments **(G)**, latency to first enter closed arms **(H)**, and distance moved during the test **(I)**. Cumulative frequency of position on the apparatus visualized in a composite heat-map **(J)**. Preference for a solution of either sucrose **(K)** or ethanol **(L)** over water. Data presented as mean ± SEM, *p < 0.05, **p < 0.01, ***p < 0.001.

Having confirmed histologically that recombination of the floxed Vmat2 exon2 takes place in Calb2 neurons, we examined if there are any phenotypic differences between cKO mice and their control littermates. Knockout mice displayed attenuated growth, increasing at relatively the same rate after 4 weeks of age (best-fit slope Ctrl 1.01 g/week, cKO 1.03 g/week), albeit consistently maintaining a weight of approximately 7 g less than control animals of the same age (Figure 6A; nonlinear regression, *p* < 0.0001 *F*_(2,370)_ = 252.1; *y*-intercept Ctrl 12.4 g, cKO 4.9 g). In the cohort of cKO mice, less than 20% did not survive past weaning age, with mortality occurring at varying postnatal time points. No premature deaths were recorded among control animals.

To evaluate basal locomotion, mice of both genotypes were placed in an open field and horizontal activity was recorded for 60 min. During this time, control animals gradually habituated, as shown by a decrease in horizontal movement. cKO mice, on the other hand, did not show habituation to the novel environment during the testing period, maintaining their initial level of movement throughout the testing period (Figure 6B; two-way RM ANOVA, the effect of genotype *p* < 0.001 *F*_(1,36)_ = 13.00; Sidak’s *post hoc* comparison Ctrl 10 *vs.* 60 min: *p* < 0.0001, cKO 10 *vs.* 60 min: *p* = 0.777). Furthermore, cKO mice spent significantly less time in the center of the arena than controls (23% *vs.* 36%, cKO *vs.* Ctrl; Student’s *t*-test, *p* < 0.01; Figure 6C).

As targeted deletion of *Vmat2* had been verified in dopamine areas controlling movement, including the SNc, mice were tested on a rotarod to assess motor coordination. cKO and control mice did not differ in their capacity to maintain their motor coordination as measured by latency to fall off during fixed speed training sessions nor at accelerating rotation speeds. For the fixed speeds, there was a negative effect on latency as a function of rotation speed (two-way RM ANOVA, the effect of speed *p* < 0.001), but no difference between genotypes (effect of genotype *p* > 0.5). For the accelerating speed test, no difference in latency to fall was observed between genotypes (Ctrl: 155.6 ± 13.4 s, cKO: 128.2 ± 16.0 s; Student’s *t*-test, *p* > 0.2; Figure 6D).

### Confounding Findings in the Elevated Plus Maze

The lack of habituation and less time spent in the center of the open field arena shown by the cKO mice prompted an examination of their response in the elevated plus maze to assess anxiety-related phenotypes. The control group spent more time in the closed arms compared to the open arms and center, as expected for this test. Surprisingly, cKO mice spent significantly more time in the open arms than controls (cKO 42%, controls 10%), with a respective decrease in time spent in closed arms (Figure 6E; two-way RM ANOVA, the effect of genotype *p* < 0.001; Sidak’s multiple comparison Ctrl *vs.* cKO open arms *p* < 0.001, closed arms *p* < 0.01). cKO mice made significantly fewer entries into the open arms (Mann–Whitney, *p* < 0.001 *U* = 74; Figure 6F), and fewer total transitions (Mann–Whitney, *p* < 0.001 *U* = 69.5; Figure 6G). Finally, cKO mice had a higher latency to the first entry of the closed arms (Student’s *t*-test, *p* < 0.001; Figure 6H), as well as lower overall locomotion (Student’s *t*-test, *p* < 0.001; Figure 6I). A composite heatmap indicating the frequency of position is presented in Figure 6J.

### Blunted Sucrose and Ethanol Preference

To study potential differences in reward processing between genotypes, we performed sucrose and ethanol preference assays. When presented with a choice between water or a sucrose solution of 1, 3, or 10%, both control and cKO mice preferred increasing concentrations of sucrose, although there was a significantly lower preference score for 10% compared to controls (Figure 6K; two-way RM ANOVA, the effect of genotype *p* < 0.05, Sidak’s *post hoc* test Ctrl *vs.* cKO 10% *p* < 0.05). When mice were tested in the ethanol preference assay for the concentration of 3, 6, and 10%, a three-way ANOVA revealed a significant effect of genotype but only a slight effect of sex (effect of genotype *p* < 0.001, the effect of sex *p* = 0.049), and this factor was thus consolidated before further analysis. From the subsequent analysis, a lower preference score for cKO mice at 6 and 10% compared to controls was observed (Figure 6L; two-way RM ANOVA, the effect of genotype *p* = 0.001, Sidak’s multiple comparison Ctrl *vs.* cKO 3% *p* = 0.5, 6% *p* < 0.001, 10% *p* < 0.01). cKO mice did not prefer ethanol over water at any concentration, scoring below 50% preference at all measuring points (Figure 6L).

### Decreased Locomotion on Repeated Doses of Amphetamine

**Figure 7:**
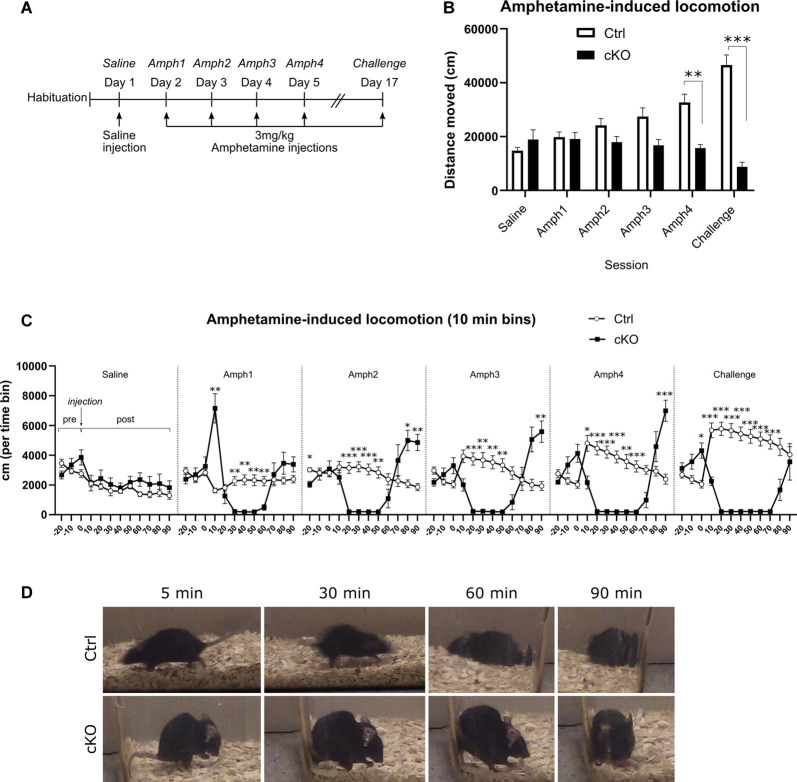
Amphetamine-induced locomotion. A sensitization paradigm **(A)** was applied to investigate the behavioral responses to amphetamine. Horizontal locomotion during 90 min following saline or amphetamine injections **(B)**. Discrete 10-min segments of each session **(C)**, including 30 min before injection (“pre”) and 90 min following injection (“post”). An example of the stereotypies exhibited at several time-points after injection of amphetamine **(D)**. Data presented as mean ± SEM, *p < 0.05, **p < 0.01, ***p < 0.001.

Amphetamine-like psychostimulants exert their physiological effects by acting, among other targets, through VMAT2. We applied an amphetamine sensitization paradigm to investigate putative differences between cKO and control mice. After receiving a saline injection on the first day, mice received daily injections of 3 mg/kg amphetamine for 4 days, followed by an interim of approximately 2 weeks, at which point the same dose was administered (“Challenge”; Figure 7A). Each session consisted of 30 min baseline recording before and 90 min of recording after i.p. injection. The dose of 3 mg/kg was selected based on previous analyses in several different VMAT2 cKO mouse lines by Isingrini et al. (2016b), which had shown that DAT-Cre mice heterozygous for *Vmat2* showed locomotor hypersensitivity to acute delivery of this particular dose. In the present study, control mice consistently increased their horizontal activity following each amphetamine administration, indicating sensitization over time (Figure 7B; Amph1 196.6 ± 20.3 m, Challenge 463.8 ± 39.4 m; two-way RM ANOVA, the effect of session *p* < 0.01, Sidak’s *post hoc* test Ctrl Amph1 *vs.* Challenge *p* < 0.001). In contrast, cKO mice did not increase their response over time but rather decreased their horizontal activity (Figure 7B; Amph1 190.8 ± 24.0 m, Challenge 87.8 ± 16.8 m, Sidak’s *post hoc* test cKO Amph1 *vs.* Challenge *p* < 0.05). Whereas the two groups moved a similar distance during the first amphetamine session (Ctrl 196.6 ± 20.3 m; cKO 190.8 ± 24.0 m; Sidak’s *post hoc* test Ctrl *vs.* cKO Amph1 *p* > 0.9), cKO mice moved significantly less during the fourth amphetamine session (Ctrl 325.1 ± 32.1 m; cKO 157.3 ± 12.7 m). Finally, during the challenge, cKO mice moved nearly only one-fifth of the distance covered by controls (Ctrl 463.8 ± 39.4 m; cKO 87.8 ± 16.8 m; two-way RM ANOVA, the effect of genotype *p* < 0.0001, Sidak’s *post hoc* test Ctrl *vs.* cKO Amph4 *p* < 0.01, Challenge *p* < 0.0001).

A closer investigation of the timeline of each session revealed numerous differences between the two genotype groups (Figure 7C). During the 30-min pre-injection period, and similarly to the basal locomotion recordings, cKO mice did not habituate to the environment as controls; rather, they tended to increase their horizontal activity during the 30 min leading up to injection (Figure 7C, “pre”-part of each session). Ten-min time-bin analysis after amphetamine injection unveiled a contrasting pattern of response between genotypes. Control mice responded to each successive injection of amphetamine by increasing initial surges of activity and gradually returning to pre-injection levels. On the other hand, after amphetamine injections, cKO mice displayed very low levels of horizontal activity, and these low-activity periods became longer during each successive session, followed by a return to, and overshoot of, the baseline activity (Figure 7C; two-way RM ANOVA, the effect of genotype *p* < 0.0001; Tukey’s *post hoc* test. Each time point is compared between genotypes. *p < 0.05, **p < 0.01 ***p < 0.001. Detailed statistics in Supplementary Table 1). During visual inspection of each session as well as a separate detailed analysis of one randomly selected case of each genotype, it became apparent that the observed immobility was the result of engagement in stereotypies, manifested mostly as gnawing of bedding (Figure 7D, bottom panels).

## Discussion

Given the importance of monoaminergic signaling in mental health and disease, and the heterogeneity of cells that compose these systems, dissecting the circuitry and physiological relevance of specific neuronal sets is paramount to provide possible mechanisms for various neuropsychiatric conditions. Here we have pursued genetic deletion of *Vmat2* selectively in Calb2-positive monoaminergic neurons by creating a new transgenic strain based on Cre-LoxP technology to address the impact of these neurons on behavioral regulation. We find varying degrees of co-localization of Calb2 and Vmat2 mRNA in fore-, mid-, and hindbrain areas: VTA, SNc, RRF, RLi, CLi, DR (mostly DRV), MnR, DRI, RMg/RIP, Rob, LC, LPGi, C1–3, and also in the arcuate nucleus. Calb2-directed Cre activity resulted in the broad but specific deletion of *Vmat2* in these same areas, which resulted in distinct phenotypic changes. Smaller in size than controls, cKO mice displayed heightened basal locomotion, anxiety-related behaviors, reduced preference for sucrose, and apparent aversion to increasing concentrations of ethanol, as well as a dramatic disengagement in locomotion following amphetamine administration. The anxiety-related behavior was not clear-cut based on the contrasting observations made in the open field and the plus maze, suggesting that further studies might be needed to fully understand this phenotype.

Taken together, the aberrant behavioral displays of the cKO mice give evidence for the substantial impact of Calb2-positive monoaminergic cells and highlight the need for specific and varied approaches to probe subpopulations. In a recently published study, the Calb2-Cre population in the VTA—representing approximately 50% of TH+ cells—was compared to another discrete VTA population defined by the transcription factor NeuroD6, which accounts for only 12% of the TH+ population in the medial aspect of the VTA (Bimpisidis et al., 2019). The Calb2 identity was further characterized as occurring in 50% of Dat (dopamine transporter)-positive cells, 7% of glutamatergic cells (Vglut2-positive), and 20% of GABAergic cells (defined by Viaat, the vesicular inhibitory amino acid transporter). Projection patterns differed for the Calb2 and NeuroD6 populations of the VTA, with fewer extra-VTA targets for the Calb2 population, and a prominent pathway from the VTA to nucleus accumbens in NeuroD6(NEX)-Cre mice. Further, optogenetic stimulation in the VTA indeed produced approach behavior for the NEX-Cre mice in a real-time place preference paradigm, while no such response was observed when Calb2-Cre mice were optogenetically stimulated in the VTA. When *Vmat2* was gene-targeted in NEX-Cre cells, using the same floxed *Vmat2* allele as here, *Vmat2^lox/lox;NEX−Cre−tg^* cKO mice, similar to control mice, displayed a preference for sucrose or ethanol over water, as well as conditioned place preference induced by amphetamine or cocaine. However, in an amphetamine sensitization paradigm, *VMAT2^Vmat2lox/lox;NEX−Cre−tg^* cKO mice showed locomotor hypersensitivity compared to controls (Bimpisidis et al., 2019; Wallén-Mackenzie, 2019). This is in stark contrast to the behavior observed in *VMAT2^lox/lox;Calb2−Cre−tg^* cKO mice studied here, following the same sensitization protocol. Thus, by comparing the phenotypes of mice in which VMAT2 has been deleted in the Calb2 *vs.* the NeuroD6 monoaminergic populations, it is clear that altering monoaminergic signaling in these two populations gives rise to very different behavioral effects. Granted, this likely stems in part from the difference in the distribution of these two genetic subpopulations—whereas NeuroD6/Vmat2 cells are present only in the VTA, Calb2/Vmat2 cells are more widely distributed in the brain, as clearly outlined in the present study.

### Histological Findings

The two-probe *in situ* hybridization approach for the detection of Vmat2 mRNA allows unambiguous identification of recombined cells. Binding of Probe1 and Probe2 indicates wildtype cells, whereas binding exclusively of Probe1 identifies cells that have undergone Cre-mediated recombination, resulting in *Vmat2* deletion. As opposed to identifying a mere “loss” of signal in KO cells, this strategy has the advantage that recombination is readily visualized and makes it possible to pinpoint the location and distribution of KO cells. Combined with a fluorescent probe for Calb2 mRNA, we were able to establish the monoaminergic identity of Calb2 cells, and show that recombination of the *Vmat2* allele was directed specifically to these cells. The data solidly confirm recombination of the floxed *Vmat2* allele in several monoaminergic populations throughout the fore-, mid- and hindbrain, more specifically in the VTA, SNc, RRF, RLi, CLi, DR (mostly DRV), MnR, DRI, RMg/RIP, Rob, LC, LPGi, C1–3, and also in the arcuate nucleus. In contrast, Vmat2 mRNA in other hypothalamic and mammillary nuclei was left unmodified. The *Vmat2^lox/lox;Calb2−Cre−tg^* cKO is thus a broad KO by targeting multiple monoaminergic systems, but specific in the sense that it is directed to Calb2-positive subpopulations within these monoaminergic systems.

Within the midbrain dopaminergic system—the VTA and SNc—our findings corroborate the approximate distributions of monoaminergic Calb2 neurons found in the rat (Rogers, 1992; Isaacs and Jacobowitz, 1994; Nemoto et al., 1999) and mouse (Liang et al., 1996; Bimpisidis et al., 2019). Among the hypothalamic dopaminergic cell groups, the limited number of Calb2/Vmat2 co-localizing cells in the arcuate nucleus concur with the literature on the rat brain (Rogers, 1992). The absence of Calb2/Vmat2 co-localization in anterior hypothalamic areas as well as the premammillary and lateral mammillary nuclei, and A13 dopaminergic groups, suggest that the Calb2-positive populations identified previously in the rat brain (Résibois and Rogers, 1992) are not monoaminergic. Importantly, beyond the dopaminergic systems, recombination was observed in various serotonergic and noradrenergic nuclei. In these areas, Calb2 mRNA was detected at varying levels, and recombination was to a high degree localized to cells expressing detectable levels of Calb2.

In the raphe nuclei, neurons with Calb2-immunoreactivity have been observed in some serotonergic subareas (Arai et al., 1991; Résibois and Rogers, 1992; Charara and Parent, 1998), although their neurotransmitter phenotype has not been established. Here, within areas such as the DR, there was clear segregation of KO cells between subareas. Surprisingly, no recombined cells were present in the DRD—corresponding to the serotonergic/dopaminergic A10dc part of the DR (Stratford and Wirtshafter, 1990)—which may have been expected given the observed colocalization of Calb2 and TH here (Rogers, 1992). On the other hand, both the DRL and, more prominently, DRV, contained numerous KO cells. Beyond neurotransmitter phenotype and projection patterns, the DR contains molecularly diverse cells (Huang et al., 2019), and further work would be needed to determine the cellular profile that Calb2/Vmat2 neurons correspond to. Additional serotonergic raphe nuclei with KO cells included the IF, raphe magnus, median raphe, and raphe obscurus. These results give evidence to the heterogeneity of the monoaminergic phenotype of Calb2 cells, which represent subpopulations within parts of the raphe.

With regards to other catecholaminergic cells, although Calb2 protein was not detected in the LC of rats (Résibois and Rogers, 1992; Rogers, 1992), it has been observed in other species (Bhagwandin et al., 2013), and mRNA is detectable in the mouse according to the Allen Brain Atlas (Lein et al., 2007). In the present study, Calb2 and Vmat2 mRNAs co-localize, and there is evident Calb2-Cre-mediated *Vmat2* recombination in the LC, the major source of noradrenaline in the brain, thus giving new evidence to Calb2 as a subpopulation marker of this nucleus. Furthermore, the adrenergic lateral paragigantocellular nucleus displayed numerous knockout cells. The results clearly show that Calb2-driven Cre expression may be used as a genetic tool to target select parts of serotonergic and catecholaminergic populations in the brain.

### Conditional Knockout Phenotype

Several of the knockout mouse lines generated to genetically target *Vmat2* preclude behavioral studies, as these transgenic models are so severely affected during development that offspring do not survive into adulthood (Takahashi et al., 1997; Wang et al., 1997; Isingrini et al., 2016b). Here, mice positive for Calb2-Cre and homozygous for *Vmat2lox* (*VMAT2^lox/lox;Calb2−Cre−tg^* cKO mice) were characterized by lower weight compared to littermates. This is consistent with several studies investigating deficiencies in monoaminergic signaling (Takahashi et al., 1997; Mooslehner et al., 2001; Narboux-Nême et al., 2013; Isingrini et al., 2016b), where serotonin seems to be critical for a postnatal growth spurt, and intact dopamine signaling necessary for survival and growth past this age. In the present study, while the growth of cKO mice is stunted, the majority survive into adulthood, with more than 80% surviving past weaning age. The observed phenotype is likely the result of a combinatorial effect of changes in dopamine and serotonin signaling, such that most mice survive, albeit never fully recovering a normal body weight. Additionally, noradrenaline signaling is crucial to postnatal survival (Ohara et al., 2013), for which the normal function of VMAT2 in Calb2 noradrenergic neurons does not appear to be crucial. Developmental changes in monoamine signaling induce neuroadaptations, and thus the structures affected by genetic targeting may have altered activity, contributing to aberrant behavior or metabolism. Among the affected areas here are the raphe obscurus, related to breathing (DePuy et al., 2011) and feeding (Wu et al., 2012); the arcuate nucleus, related to several aspects of homeostasis; the LC, related to arousal, sympathetic regulation, and responses to stress as well as cognitive functions (reviewed by Chandler, 2016); as well as the VTA and SNc, related to motivation, reward-related behavior, and movement (Schultz, 1998; Salamone and Correa, 2012; Howe and Dombeck, 2016). Consequently, to address the impact of these cellular changes on behavior, multiple tests were performed to characterize the cKO phenotype.

### Conflicting Aspects of Anxiety and Stress-Related Parameters

When placed in a novel environment, cKO mice displayed impaired habituation, suggesting an anxious-like phenotype. Less time spent in the center of the arena than controls supports this finding, arguing against the increased exploratory drive. The elevated plus maze is an established method to study anxiety in rodents, recently validated in a virtual-reality test adapted to humans (Biedermann et al., 2017), where avoidance of open arms measured as time spent is a correlate of anxiety. When tested in the elevated plus maze, cKO mice spent more time in the open arms compared to controls, indicating an anxiolytic phenotype. However, there were important observations during this test that affect this interpretation. cKO mice demonstrated high latency to first entering the closed arms, lower number of entries made into the open arms, and less total movement during the test. The immobility of cKO mice could be interpreted as increased anxiety (Pellow et al., 1985; Walf and Frye, 2007), corroborating the behavior seen in the novel open field test. The results are similar to those seen for a serotonin system-specific knockout of VMAT2 (*VMAT2^lox/lox;SERT-Cre^*, Narboux-Nême et al., 2011), suggesting this observation could be a serotonergic effect. A more recent study using the elevated plus maze showed no change in heterozygous *VMAT2^lox/wtx;SERT−Cre^* mice compared to controls (Isingrini et al., 2016b). Thus, our results indicate that the observed behavioral change stems from a dysfunction in a specific set of neurons rather than a global decrease in serotonin. However, beyond this hypothesis, a firmer validation would require more advanced experimental tools.

For constitutive VMAT2 heterozygous animals, a change in depressive-like phenotype as measured by a decreased preference for sucrose solution has been reported, without concurrent changes in anxiety (Fukui et al., 2007). Similarly, in the present study, cKO mice show a somewhat blunted preference for the sucrose solution—indicative of anhedonia—but show equivocal anxiety-related responses. The possibility of increased freezing responses related to stress specifically in the elevated plus maze remains. Thus, further tests are required to disentangle anxiety, stress, and/or motor-related phenotypes in the cKO mice.

### Reduced Ethanol Intake

Monoamine signaling has been linked to several neuropsychiatric conditions, and alterations in these systems may contribute to predisposition to addictions including alcoholism, for which certain variations in the promoter region of the human VMAT2 are indicated as protective factors (Lin et al., 2005). VMAT2 deficient heterozygotes are hypersensitive to the locomotor effects of ethanol (Wang et al., 1997) and show a decreased preference toward ethanol in a two-bottle test (males) as well as diminished conditioned place preference (Savelieva et al., 2006). In the present study, cKO mice did not display a preference for any concentration of ethanol. Rather, a preference score below 50% at all points measured with a tendency to decrease with increasing ethanol concentration suggests a possible aversion. However, to clearly demonstrate aversion, more specific behavioral tests would be needed, such as conditioned taste aversion. It is unknown for instance if the behavior seen here is a consequence of an aversion to the pharmacological effects or taste of the ethanol. Further, it is unknown whether this effect is caused by a global decrease of neurotransmitter or *via* a functional change specifically in the Calb2/Vmat2 cells. Of note, however, is that the phenomenon is presented in both sexes, rather than isolated to males as for VMAT2 heterozygotes (Savelieva et al., 2006). Cellular recordings of VTA neurons in response to ethanol show a preferential response in medial regions (Mrejeru et al., 2015), and several other regional differences in sensitivity have been indicated. Thus, this population of Calb2 VTA neurons is a potential tool to investigate reward-related responses to ethanol. Finally, the CLi, which here showed extensive recombination of the *Vmat2* gene, has been implicated as a key structure mediating alcohol preference (Dudek and Hyytiä, 2016) and nicotine self-infusion (Ikemoto et al., 2006), further warranting inquiry in the particular role of Calb2-positive monoaminergic neurons in this region. Thus, although direct optogenetic stimulation of Calb2-Cre neurons in the VTA did not result in increased approach behavior in an optogenetic real-time place preference paradigm (Bimpisidis et al., 2019), the present results do tentatively indicate a potential role of these cells in reward-processing.

### Motor Activity and Response to Amphetamine

While some results indicate that constitutive *Vmat2* heterozygote mice show no change in basal locomotion (Takahashi et al., 1997), others have shown a decrease in spontaneous activity (Fukui et al., 2007), appears to be specifically related to a reduction of VMAT2 in dopamine transporter (DAT)-Cre-positive cells (Isingrini et al., 2016b). Here, *VMAT2^lox/lox;Calb2−Cre−tg^* animals display heightened basal locomotor activity, exemplified by a lack of habituation. During the rotarod task, no difference in motor coordination was detected at different fixed speeds, similar to observations of constitutive VMAT2 heterozygotes (Takahashi et al., 1997).

Different aspects of monoamine signaling have been identified as contributing to the locomotor response to drugs. For instance, altering dopamine signaling is sufficient to alter the motor response to acute amphetamine, while the same relationship was observed between the serotonin system and cocaine (Isingrini et al., 2016b). Here, there is most likely involvement of several of the transmitter systems, although to a more restricted extent than complete knockout of any one system. The decrease in locomotor activity on repeated injection of amphetamine may be explained by increased sensitivity to and engagement in stereotypical behaviors, similar to previous observations in *Vmat2* heterozygotes (Wang et al., 1997) and so-called VMAT-LO mice, which have a hypomorphic *Vmat2* allele resulting in a 95% reduction of VMAT2 (Mooslehner et al., 2001). A possible mechanism is a developmental compensatory oversensitivity to dopamine, which exacerbates the behavioral response to the extra dopamine released on amphetamine stimulation. Importantly, this has previously been linked to neurochemical changes in the direct and indirect output of striatal areas (Mooslehner et al., 2001), and lesions of the nigrostriatal pathway (Fibiger et al., 1973) and the patch compartment of the striatum (Murray et al., 2015) attenuate stereotypy induced by amphetamine and cocaine respectively. As previously observed for VMAT2 heterozygous mice (Wang et al., 1997), the response to the first amphetamine session here was, in fact, greater for cKO mice than controls, while lower in subsequent sessions. This is in keeping with increased engagement in stereotypes following chronic administration of amphetamine (Segal and Mandell, 1974), supporting the hypothesis of neuroadaptive changes. As observed in previous studies (Fon et al., 1997; Takahashi et al., 1997; Wang et al., 1997; Isingrini et al., 2016b, 2017), dopamine seems to be produced at a normal rate in cells lacking VMAT2 but metabolized more quickly, overall decreasing cellular content. Thus, although amphetamine elicits the non-physiological release of dopamine by reversal of dopamine transporter from cells lacking VMAT2, it is likely that sensitization of output areas plays a greater role in explaining the response. Additionally, given the sites of KO cells including the LC, the behavioral output seen here likely stems from effects exerted by other signaling systems, notably that of noradrenaline signaling, which has considerable interactions with dopaminergic signaling with relevance to the response to stimulants (Ferrucci et al., 2019), and has been implicated in sensitivity to stereotypies (Weinshenker et al., 2002).

### Further Directions

Among the areas with an extensive knockout of VMAT2 were the raphe magnus and lateral paragigantocellular nuclei. *Via* distinct projections to the spinal cord, these may have different modulatory effects on nociceptive signaling (Condés-Lara et al., 2012; Gautier et al., 2017). Although it is known that monoamine signaling affects pain signaling, it is hitherto unknown whether VMAT2 deficiency is of any consequence for pain sensitivity. Other brain regions implicated in pain control include the LC (reviewed by Taylor and Westlund, 2017), further warranting investigation of the role of altered VMAT2 function given the histological findings presented here.

In addition to specific tests as presented here, phenotyping of transgenic mouse lines can be performed by home-cage monitoring, which is gaining increasing attention in research methodology. This would enable collecting data on such parameters as wake/sleep patterns, feeding behaviors, and social interactions, allowing further characterization of the knockout line (Richardson, 2015; Balzani et al., 2018; Pernold et al., 2019). Considering the major roles played by monoaminergic neurons, this type of combined analysis in the home-cage environment would likely enhance the understanding of the phenotype obtained when deleting VMAT2 selectively from the Calb2-Cre population of neurons.

#### Limitations

When assessing the effects of a cKO of any gene, it is relevant to point out that developmental neuroadaptations may have taken place. This is important not least in the current context since both *Calb2* and *Vmat2* genes, i.e., both the driver and the effector, are induced early on in embryonal development (Dumas and Wallén-Mackenzie, 2019). Thus, it is not possible to conclude as to when the herein reported phenotypes are established, other than that they are present in the adult mouse.

Further, while the present study aimed at characterizing the distribution of Calb2+/Vmat2+ populations in the brain, and also address their significance in behavior by genetic targeting of the *Vmat2* gene selectively in the Calb2-positive subtype of monoaminergic neurons, no analysis of neurotransmitter release or the expected down-regulation of VMAT2 protein levels were performed. This is a caveat of the present study. Other studies have indeed reported these types of analyses (Fon et al., 1997; Takahashi et al., 1997; Wang et al., 1997; Mooslehner et al., 2001; Narboux-Nême et al., 2011; Isingrini et al., 2016b) while the present study lacked this focus and hence, certain mechanistic conclusions cannot be drawn.

For many of the parameters studied, crosstalk on molecular and cellular levels between the different monoamine systems exist—for instance, locomotor response to cocaine has been suggested to be exaggerated due to potentiation of the 5HT1A receptor (Szumlinski et al., 2004), and indeed diverging results were found depending on the involvement of one or several monoamine systems (Isingrini et al., 2016b). At the network level, the transmission of neurotransmitters has cross-modulatory effects, such as described for dopamine and noradrenaline (Smith and Greene, 2012; Ferrucci et al., 2019). Considering the above-mentioned limitations of the present study, pharmacological interventions or the site-specific introduction of shRNA targeting *Vmat2* in adult Calb2-Cre mice may clarify the involvement of each region and corresponding transmitter system, as well as avoid developmental effects of a constitutive knockout.

### Conclusion

Taken together, the present findings contribute to the observations that disrupted monoaminergic signaling in neurons defined and joined by their expression of the *Calb2* gene plays important roles in diverse facets of behavior, and provides new evidence for the presence and distribution of Calb2 mRNA in monoaminergic brain regions. Site-specific and adult interventions targeting selected Calb2-positive monoaminergic populations would allow delineation of the precise role of these neurons.

## Data Availability Statement

The raw data supporting the conclusions of this article will be made available by the authors, without undue reservation.

## Ethics Statement

The animal study was reviewed and approved by Uppsala Ethical Committee for Laboratory Animal Research (Uppsala djurförsöksetiska nämnd Uppsala Tingsrätt Box 1113 751 41 Uppsala).

## Author Contributions

ÅW-M conceived the study and was in charge of overall planning. NK, ZB, SD, and ÅW-M designed and performed research, and analyzed data. ÅW-M, NK, and ZB wrote the manuscript. All authors contributed to the article and approved the submitted version.

## Conflict of Interest

SD is the owner of Oramacell. The remaining authors declare that the research was conducted in the absence of any commercial or financial relationships that could be construed as a potential conflict of interest.

## References

[B1] Arai R. Winsky L. Arai M. Jacobowitz D. M. (1991). Immunohistochemical localization of calretinin in the rat hindbrain. J. Comp. Neurol. 310, 21–44. 10.1002/cne.903100105 1939729

[B2] Balzani E. Falappa M. Balci F. Tucci V. (2018). An approach to monitoring home-cage behavior in mice that facilitates data sharing. Nat. Protoc. 13, 1331–1347. 10.1038/nprot.2018.031 29773907

[B3] Barinka F. Druga R. (2010). Calretinin expression in the mammalian neocortex: a review. Physiol. Res. 59, 665–677. 20406030 10.33549/physiolres.931930

[B4] Bhagwandin A. Gravett N. Bennett N. C. Manger P. R. (2013). Distribution of parvalbumin, calbindin and calretinin containing neurons and terminal networks in relation to sleep associated nuclei in the brain of the giant Zambian mole-rat (*Fukomys mechowii*). J. Chem. Neuroanat. 52, 69–79. 10.1016/j.jchemneu.2013.06.002 23796985

[B5] Biedermann S. V. Biedermann D. G. Wenzlaff F. Kurjak T. Nouri S. Auer M. K.. (2017). An elevated plus-maze in mixed reality for studying human anxiety-related behavior. BMC Biol. 15:125. 10.1186/s12915-017-0463-6 29268740 PMC5740602

[B6] Bimpisidis Z. König N. Stagkourakis S. Zell V. Vlcek B. Dumas S.. (2019). The NeuroD6 subtype of VTA neurons contributes to psychostimulant sensitization and behavioral reinforcement. eNeuro 6:ENEURO.0066-19.2019. 10.1523/ENEURO.0066-19.2019 31097625 PMC6565376

[B7] Bimpisidis Z. König N. Wallén-Mackenzie Å. (2020). Two different real-time place preference paradigms using optogenetics within the ventral tegmental area of the mouse. J. Vis. Exp. 156:e60867. 10.3791/60867 32116305

[B8] Chandler D. J. (2016). Evidence for a specialized role of the locus coeruleus noradrenergic system in cortical circuitries and behavioral operations. Brain Res. 1641, 197–206. 10.1016/j.brainres.2015.11.022 26607255 PMC4879003

[B9] Charara A. Parent A. (1998). Chemoarchitecture of the primate dorsal raphe nucleus. J. Chem. Neuroanat. 15, 111–127. 10.1016/s0891-0618(98)00036-2 9719363

[B10] Christiansen L. Tan Q. Iachina M. Bathum L. Kruse T. A. McGue M.. (2007). Candidate gene polymorphisms in the serotonergic pathway: influence on depression symptomatology in an elderly population. Biol. Psychiatry 61, 223–230. 10.1016/j.biopsych.2006.03.046 16806099

[B11] Condés-Lara M. Rojas-Piloni G. Martínez-Lorenzana G. Diez-Martínez D. C. Rodríguez-Jiménez J. (2012). Functional interactions between the paraventricular hypothalamic nucleus and raphe magnus. A comparative study of an integrated homeostatic analgesic mechanism. Neuroscience 209, 196–207. 10.1016/j.neuroscience.2012.02.032 22390942

[B12] Cools R. (2008). Role of dopamine in the motivational and cognitive control of behavior. Neuroscientist 14, 381–395. 10.1177/1073858408317009 18660464

[B13] Cools R. Roberts A. C. Robbins T. W. (2008). Serotoninergic regulation of emotional and behavioural control processes. Trends Cogn. Sci. 12, 31–40. 10.1016/j.tics.2007.10.011 18069045

[B14] DePuy S. D. Kanbar R. Coates M. Stornetta R. L. Guyenet P. G. (2011). Control of breathing by raphe obscurus serotonergic neurons in mice. J. Neurosci. 31, 1981–1990. 10.1523/JNEUROSCI.4639-10.2011 21307236 PMC3071248

[B15] Dudek M. Hyytiä P. (2016). Alcohol preference and consumption are controlled by the caudal linear nucleus in alcohol-preferring rats. Eur. J. Neurosci. 43, 1440–1448. 10.1111/ejn.13245 27038036

[B16] Dumas S. Wallén-Mackenzie Å. (2019). Developmental co-expression of Vglut2 and Nurr1 in a Mes-Di-encephalic continuum preceeds dopamine and glutamate neuron specification. Front. Cell Dev. Biol. 7:307. 10.3389/fcell.2019.00307 31850343 PMC6892754

[B17] Fairless R. Williams S. K. Diem R. (2019). Calcium-binding proteins as determinants of central nervous system neuronal vulnerability to disease. Int. J. Mol. Sci. 20:2146. 10.3390/ijms20092146 31052285 PMC6539299

[B18] Ferrucci M. Limanaqi F. Ryskalin L. Biagioni F. Busceti C. L. Fornai F. (2019). The effects of amphetamine and methamphetamine on the release of norepinephrine, Dopamine and acetylcholine from the brainstem reticular formation. Front. Neuroanat. 13:48. 10.3389/fnana.2019.00048 31133823 PMC6524618

[B19] Fibiger H. C. Fibiger H. P. Zis A. P. (1973). Attenuation of amphetamine-induced motor stimulation and stereotypy by 6-hydroxydopamine in the rat. Br. J. Pharmacol. 47, 683–692 10.1111/j.1476-5381.1973.tb08194.x 4146741 PMC1776066

[B20] Fields H. L. Hjelmstad G. O. Margolis E. B. Nicola S. M. (2007). Ventral tegmental area neurons in learned appetitive behavior and positive reinforcement. Annu. Rev. Neurosci. 30, 289–316. 10.1146/annurev.neuro.30.051606.094341 17376009

[B21] Fon E. A. Pothos E. N. Sun B. C. Killeen N. Sulzer D. Edwards R. H. (1997). Vesicular transport regulates monoamine storage and release but is not essential for amphetamine action. Neuron 19, 1271–1283. 10.1016/s0896-6273(00)80418-3 9427250

[B22] Fortin M. Parent A. (1996). Calretinin as a marker of specific neuronal subsets in primate substantia nigra and subthalamic nucleus. Brain Res. 708, 201–204. 10.1016/0006-8993(95)01374-1 8720880

[B23] Fukui M. Rodriguiz R. M. Zhou J. Jiang S. X. Phillips L. E. Caron M. G.. (2007). Vmat2 heterozygous mutant mice display a depressive-like phenotype. J. Neurosci. 27, 10520–10529. 10.1523/JNEUROSCI.4388-06.2007 17898223 PMC2855647

[B24] Gautier A. Geny D. Bourgoin S. Bernard J. F. Hamon M. (2017). Differential innervation of superficial versus deep laminae of the dorsal horn by bulbo-spinal serotonergic pathways in the rat. IBRO Rep. 2, 72–80. 10.1016/j.ibror.2017.04.001 30135935 PMC6084826

[B25] Guillot T. S. Miller G. W. (2009). Protective actions of the vesicular monoamine transporter 2 (VMAT2) in monoaminergic neurons. Mol. Neurobiol. 39, 149–170. 10.1007/s12035-009-8059-y 19259829

[B26] Gutiérrez B. Rosa A. Papiol S. Arrufat F. J. Catalán R. Salgado P.. (2007). Identification of two risk haplotypes for schizophrenia and bipolar disorder in the synaptic vesicle monoamine transporter gene (SVMT). Am. J. Med. Genet. B Neuropsychiatr. Genet. 144B, 502–507. 10.1002/ajmg.b.30499 17427184

[B27] Haas H. L. Sergeeva O. A. Selbach O. (2008). Histamine in the nervous system. Physiol. Rev. 88, 1183–1241. 10.1152/physrev.00043.2007 18626069

[B28] Howe M. W. Dombeck D. A. (2016). Rapid signalling in distinct dopaminergic axons during locomotion and reward. Nature 535, 505–510. 10.1038/nature18942 27398617 PMC4970879

[B29] Huang K. W. Ochandarena N. E. Philson A. C. Hyun M. Birnbaum J. E. Cicconet M.. (2019). Molecular and anatomical organization of the dorsal raphe nucleus. eLife 8:e46464. 10.7554/eLife.46464 31411560 PMC6726424

[B30] Ikemoto S. Qin M. Liu Z.-H. (2006). Primary reinforcing effects of nicotine are triggered from multiple regions both inside and outside the ventral tegmental area. J. Neurosci. 26, 723–730. 10.1523/JNEUROSCI.4542-05.2006 16421292 PMC1380251

[B31] Isaacs K. R. Jacobowitz D. M. (1994). Mapping of the colocalization of calretinin and tyrosine hydroxylase in the rat substantia nigra and ventral tegmental area. Exp. Brain Res. 99, 34–42. 10.1007/BF00241410 7925794

[B32] Isingrini E. Guinaudie C. Perret L. C. Rainer Q. Moquin L. Gratton A.. (2017). Genetic elimination of dopamine vesicular stocks in the nigrostriatal pathway replicates Parkinson’s disease motor symptoms without neuronal degeneration in adult mice. Sci. Rep. 7:12432. 10.1038/s41598-017-12810-9 28963508 PMC5622135

[B33] Isingrini E. Perret L. Rainer Q. Amilhon B. Guma E. Tanti A.. (2016a). Resilience to chronic stress is mediated by noradrenergic regulation of dopamine neurons. Nat. Neurosci. 19, 560–563. 10.1038/nn.4245 26878672

[B34] Isingrini E. Perret L. Rainer Q. Sagueby S. Moquin L. Gratton A.. (2016b). Selective genetic disruption of dopaminergic, serotonergic and noradrenergic neurotransmission: insights into motor, emotional and addictive behaviour. J. Psychiatry Neurosci. 41, 169–181. 10.1503/jpn.150028 26505143 PMC4853208

[B35] Keiflin R. Janak P. H. (2015). Dopamine prediction errors in reward learning and addiction: from theory to neural circuitry. Neuron 88, 247–263. 10.1016/j.neuron.2015.08.037 26494275 PMC4760620

[B36] Lein E. S. Hawrylycz M. J. Ao N. Ayres M. Bensinger A. Bernard A.. (2007). Genome-wide atlas of gene expression in the adult mouse brain. Nature 445, 168–176. 10.1038/nature05453 17151600

[B37] Liang C.-L. Sinton C. M. German D. C. (1996). Midbrain dopaminergic neurons in the mouse: co-localization with Calbindin-D28k and calretinin. Neuroscience 75, 523–533. 10.1016/0306-4522(96)00228-x 8931015

[B38] Likhtik E. Johansen J. P. (2019). Neuromodulation in circuits of aversive emotional learning. Nat. Neurosci. 22, 1586–1597. 10.1038/s41593-019-0503-3 31551602

[B39] Lin Z. Walther D. Yu X.-Y. Li S. Drgon T. Uhl G. R. (2005). SLC18A2 promoter haplotypes and identification of a novel protective factor against alcoholism. Hum. Mol. Genet. 14, 1393–1404. 10.1093/hmg/ddi148 15829504

[B40] Lohr K. M. Chen M. Hoffman C. A. McDaniel M. J. Stout K. A. Dunn A. R.. (2016). Vesicular monoamine transporter 2 (VMAT2) level regulates MPTP vulnerability and clearance of excess dopamine in mouse striatal terminals. Toxicol. Sci. 153, 79–88. 10.1093/toxsci/kfw106 27287315 PMC5013878

[B41] McRitchie D. A. Hardman C. D. Halliday G. M. (1996). Cytoarchitectural distribution of calcium binding proteins in midbrain dopaminergic regions of rats and humans. J. Comp. Neurol. 364, 121–150. 10.1002/(SICI)1096-9861(19960101)364:1<121::AID-CNE11>3.0.CO;2-1 8789281

[B42] Mongia S. Yamaguchi T. Liu B. Zhang S. Wang H. Morales M. (2019). The Ventral Tegmental Area has calbindin neurons with the capability to co-release glutamate and dopamine into the nucleus accumbens. Eur. J. Neurosci. 50, 3968–3984. 10.1111/ejn.14493 31215698 PMC6920608

[B43] Mooslehner K. A. Chan P. M. Xu W. Liu L. Smadja C. Humby T.. (2001). Mice with very low expression of the vesicular monoamine transporter 2 gene survive into adulthood: potential mouse model for Parkinsonism. Mol. Cell. Biol. 21, 5321–5331. 10.1128/MCB.21.16.5321-5331.2001 11463816 PMC87256

[B44] Mouatt-Prigent A. Agid Y. Hirsch E. C. (1994). Does the calcium binding protein calretinin protect dopaminergic neurons against degeneration in Parkinson’s disease? Brain Res. 668, 62–70. 10.1016/0006-8993(94)90511-8 7704619

[B45] Mrejeru A. Martí-Prats L. Avegno E. M. Harrison N. L. Sulzer D. (2015). A subset of ventral tegmental area dopamine neurons responds to acute ethanol. Neuroscience 290, 649–658. 10.1016/j.neuroscience.2014.12.081 25660505 PMC4587983

[B46] Murray R. C. Logan M. C. Horner K. A. (2015). Striatal patch compartment lesions reduce stereotypy following repeated cocaine administration. Brain Res. 1618, 286–298. 10.1016/j.brainres.2015.06.012 26100338 PMC4522223

[B47] Narboux-Nême N. Angenard G. Mosienko V. Klempin F. Pitychoutis P. M. Deneris E.. (2013). Postnatal growth defects in mice with constitutive depletion of central serotonin. ACS Chem. Neurosci. 4, 171–181. 10.1021/cn300165x 23336056 PMC3547491

[B48] Narboux-Nême N. Sagné C. Doly S. Diaz S. L. Martin C. B. P. Angenard G.. (2011). Severe serotonin depletion after conditional deletion of the vesicular monoamine transporter 2 gene in serotonin neurons: neural and behavioral consequences. Neuropsychopharmacology 36, 2538–2550. 10.1038/npp.2011.142 21814181 PMC3194080

[B49] Nemoto C. Hida T. Arai R. (1999). Calretinin and calbindin-D28k in dopaminergic neurons of the rat midbrain: a triple-labeling immunohistochemical study. Brain Res. 846, 129–136. 10.1016/s0006-8993(99)01950-2 10536220

[B50] Ng J. Papandreou A. Heales S. J. Kurian M. A. (2015). Monoamine neurotransmitter disorders—clinical advances and future perspectives. Nat. Rev. Neurol. 11, 567–584. 10.1038/nrneurol.2015.172 26392380

[B51] Ohara A. Kasahara Y. Yamamoto H. Hata H. Kobayashi H. Numachi Y.. (2013). Exclusive expression of VMAT2 in noradrenergic neurons increases viability of homozygous VMAT2 knockout mice. Biochem. Biophys. Res. Commun. 432, 526–532. 10.1016/j.bbrc.2013.02.014 23410751

[B52] Padmakumar M. Jaeken J. Ramaekers V. Lagae L. Greene D. Thys C.. (2019). A novel missense variant in SLC18A2 causes recessive brain monoamine vesicular transport disease and absent serotonin in platelets. JIMD Rep. 47, 9–16. 10.1002/jmd2.12030 31240161 PMC6498820

[B53] Pellow S. Chopin P. File S. E. Briley M. (1985). Validation of open: closed arm entries in an elevated plus-maze as a measure of anxiety in the rat. J. Neurosci. Methods 14, 149–167. 10.1016/0165-0270(85)90031-7 2864480

[B54] Pernold K. Iannello F. Low B. E. Rigamonti M. Rosati G. Scavizzi F.. (2019). Towards large scale automated cage monitoring—diurnal rhythm and impact of interventions on in-cage activity of C57BL/6J mice recorded 24/7 with a non-disrupting capacitive-based technique. PLoS One 14:e0211063. 10.1371/journal.pone.0211063 30716111 PMC6361443

[B55] Poulin J.-F. Zou J. Drouin-Ouellet J. Kim K.-Y. A. Cicchetti F. Awatramani R. B. (2014). Defining midbrain dopaminergic neuron diversity by single-cell gene profiling. Cell Rep. 9, 930–943. 10.1016/j.celrep.2014.10.008 25437550 PMC4251558

[B56] Résibois A. Rogers J. H. (1992). Calretinin in rat brain: an immunohistochemical study. Neuroscience 46, 101–134. 10.1016/0306-4522(92)90012-q 1594096

[B57] Richardson C. A. (2015). The power of automated behavioural homecage technologies in characterizing disease progression in laboratory mice: a review. Appl. Anim. Behav. Sci. 163, 19–27. 10.1016/j.applanim.2014.11.018

[B58] Rilstone J. J. Alkhater R. A. Minassian B. A. (2013). Brain dopamine-serotonin vesicular transport disease and its treatment. N. Engl. J. Med. 368, 543–550. 10.1056/NEJMoa1207281 23363473

[B59] Rogers J. H. (1992). Immunohistochemical markers in rat brain: colocalization of calretinin and calbindin-D28k with tyrosine hydroxylase. Brain Res. 587, 203–210. 10.1016/0006-8993(92)90998-o 1356063

[B60] Salamone J. D. Correa M. (2012). The mysterious motivational functions of mesolimbic dopamine. Neuron 76, 470–485. 10.1016/j.neuron.2012.10.021 23141060 PMC4450094

[B61] Sara S. J. Bouret S. (2012). Orienting and reorienting: the locus coeruleus mediates cognition through arousal. Neuron 76, 130–141. 10.1016/j.neuron.2012.09.011 23040811

[B62] Savelieva K. V. Caudle W. M. Miller G. W. (2006). Altered ethanol-associated behaviors in vesicular monoamine transporter heterozygote knockout mice. Alcohol 40, 87–94. 10.1016/j.alcohol.2006.09.030 17307644

[B63] Schuldiner S. Shirvan A. Linial M. (1995). Vesicular neurotransmitter transporters: from bacteria to humans. Physiol. Rev. 75, 369–392. 10.1152/physrev.1995.75.2.369 7724667

[B64] Schultz W. (1998). Predictive reward signal of dopamine neurons. J. Neurophysiol. 80, 1–27. 10.1152/jn.1998.80.1.1 9658025

[B65] Schultz W. Studer A. Romo R. Sundstrom E. Jonsson G. Scarnati E. (1989). Deficits in reaction times and movement times as correlates of hypokinesia in monkeys with MPTP-induced striatal dopamine depletion. J. Neurophysiol. 61, 651–668. 10.1152/jn.1989.61.3.651 2785168

[B66] Schwaller B. (2014). Calretinin: from a “simple” Ca^2+^ buffer to a multifunctional protein implicated in many biological processes. Front. Neuroanat. 8:3. 10.3389/fnana.2014.00003 24550787 PMC3913827

[B67] Segal D. S. Mandell A. J. (1974). Long-term administration of d-amphetamine: progressive augmentation of motor activity and stereotypy. Pharmacol. Biochem. Behav. 2, 249–255. 10.1016/0091-3057(74)90060-4 4857295

[B68] Smith C. C. Greene R. W. (2012). CNS dopamine transmission mediated by noradrenergic innervation. J. Neurosci. 32, 6072–6080. 10.1523/JNEUROSCI.6486-11.2012 22553014 PMC3371362

[B69] Stratford T. R. Wirtshafter D. (1990). Ascending dopaminergic projections from the dorsal raphe nucleus in the rat. Brain Res. 511, 173–176. 10.1016/0006-8993(90)90239-8 1970510

[B70] Sulzer D. Sonders M. S. Poulsen N. W. Galli A. (2005). Mechanisms of neurotransmitter release by amphetamines: a review. Prog. Neurobiol. 75, 406–433. 10.1016/j.pneurobio.2005.04.003 15955613

[B71] Szumlinski K. K. Frys K. A. Kalivas P. W. (2004). Dissociable roles for the dorsal and median raphé in the facilitatory effect of 5-HT1A receptor stimulation upon cocaine-induced locomotion and sensitization. Neuropsychopharmacology 29, 1675–1687. 10.1038/sj.npp.1300473 15127081

[B72] Takahashi N. Miner L. L. Sora I. Ujike H. Revay R. S. Kostic V.. (1997). VMAT2 knockout mice: heterozygotes display reduced amphetamine-conditioned reward, enhanced amphetamine locomotion and enhanced MPTP toxicity. Proc. Natl. Acad. Sci. U S A 94, 9938–9943. 10.1073/pnas.94.18.9938 9275230 PMC23302

[B73] Taylor B. K. Westlund K. N. (2017). The noradrenergic locus coeruleus as a chronic pain generator. J. Neurosci. Res. 95, 1336–1346. 10.1002/jnr.23956 27685982 PMC5374049

[B74] Viereckel T. Dumas S. Smith-Anttila C. J. A. Vlcek B. Bimpisidis Z. Lagerström M. C.. (2016). Midbrain gene screening identifies a new mesoaccumbal glutamatergic pathway and a marker for dopamine cells neuroprotected in Parkinson’s disease. Sci. Rep. 6:35203. 10.1038/srep35203 27762319 PMC5071886

[B75] Walf A. A. Frye C. A. (2007). The use of the elevated plus maze as an assay of anxiety-related behavior in rodents. Nat. Protoc. 2, 322–328. 10.1038/nprot.2007.44 17406592 PMC3623971

[B76] Wallén-Mackenzie Å. (2019). Who Does What in the Heterogeneous VTA? Spotlight on the Newly Identified NeuroD6 VTA Subtype. Available online at: https://neuronline.sfn.org/scientific-research/who-does-what-in-the-heterogeneous-vta-spotlight-on-the-newly-identified-neurod6-vta-subtype. Accessed June 30, 2020.

[B77] Wang Y.-M. Gaidetdinov R. R. Fumagalli F. Xu F. Jones S. R. Bock C. B.. (1997). Knockout of the vesicular monoamine transporter 2 gene results in neonatal death and supersensitivity to cocaine and amphetamine. Neuron 19, 1285–1296. 10.1016/s0896-6273(00)80419-5 9427251

[B78] Weinshenker D. Miller N. S. Blizinsky K. Laughlin M. L. Palmiter R. D. (2002). Mice with chronic norepinephrine deficiency resemble amphetamine-sensitized animals. Proc. Natl. Acad. Sci. U S A 99, 13873–13877. 10.1073/pnas.212519999 12370425 PMC129790

[B79] Wu Q. Clark M. S. Palmiter R. D. (2012). Deciphering a neuronal circuit that mediates appetite. Nature 483, 594–597. 10.1038/nature10899 22419158 PMC4000532

[B80] Xu Y. Y. Lu Y. Xu P. Mangieri L. R. Isingrini E. Xu Y. Y.. (2017). VMAT2-mediated neurotransmission from midbrain leptin receptor neurons in feeding regulation. eNeuro 4:ENEURO.0083–17.2017. 10.1523/ENEURO.0083-17.2017 28560316 PMC5446488

