# Supplementary material for: Selective Knockout of the Vesicular Monoamine Transporter 2 (Vmat2) Gene in Calbindin2/Calretinin-Positive Neurons Results in Profound Changes in Behavior and Response to Drugs of Abuse
Source: Front Behav Neurosci. 2020 Nov 9;14:578443. doi: 10.3389/fnbeh.2020.578443 (PMC7680758; doi:10.3389/fnbeh.2020.578443)
Supplement: Supplementary file 1 [file Data_Sheet_1.PDF]

Supplementary material

Figure S1 – Radioactive *in situ* hybridization

Table S1 – Detailed statistics for Figure 7 C

Figure S1

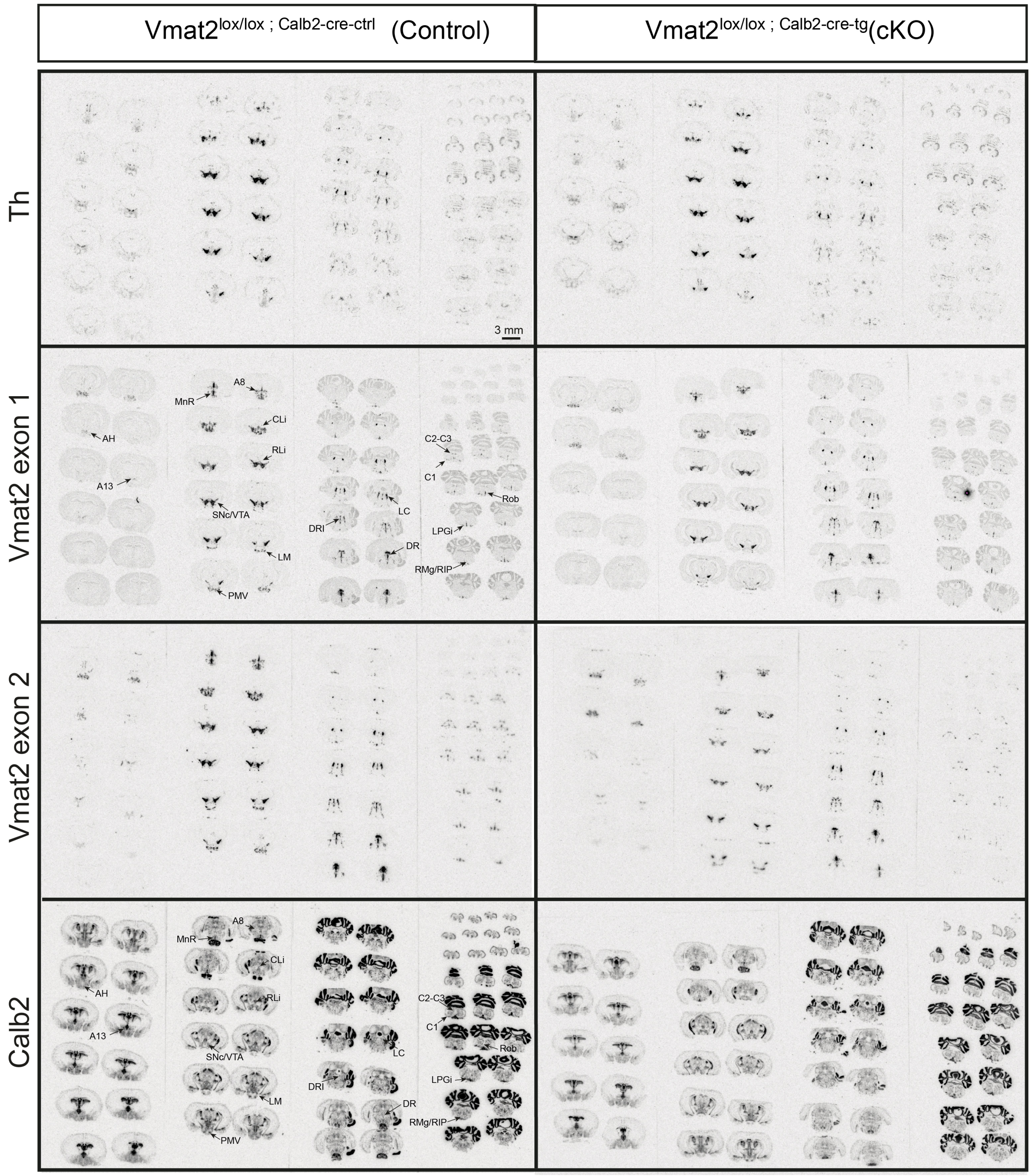

Figure S1. Radioisotope labeled *in situ* hybridization for detection of Th, Vmat2 exon 1, Vmat2 exon 2, and Calb2 mRNA. AH, anterior hypothalamus; Cli, caudal linear nucleus; DRI, interfascicular dorsal raphe; LC, locus coeruleus; LM, lateral mammillary nucleus; LPGi, lateral paragigantocellular nucleus; MnR, median raphe; PMV, ventral premammillary nucleus; Rob, raphe obscurus; RLi, rostral linear nucleus; RMg/RIP, raphe magnus/raphe interpositus; RRF, retrorubral field; SNc, substantia nigra compacta; Th, tyrosine hydroxylase; VTA, ventral tegmental area.

Table S1. Analysis of 10-minute time bins of amphetamine-induced locomotion. Multiple comparison statistics for Figure 6 C.

Timepoint	Tukey's multiple comparisons test	Mean Diff.	95.00% CI of diff.	Significant?	Summary	Adjusted P Value
-20	Ctrl saline vs. cKO saline	785.7	-614.2 to 2186	No	ns	0.6535
-10	Ctrl saline vs. cKO saline	-410.9	-2227 to 1405	No	ns	0.9987
0	Ctrl saline vs. cKO saline	-1085	-3207 to 1037	No	ns	0.7395
10	Ctrl saline vs. cKO saline	-31.43	-2158 to 2095	No	ns	>0.9999
20	Ctrl saline vs. cKO saline	-543.7	-2874 to 1786	No	ns	0.998
30	Ctrl saline vs. cKO saline	-432.5	-2443 to 1577	No	ns	0.9995
40	Ctrl saline vs. cKO saline	-223.3	-1568 to 1121	No	ns	>0.9999
50	Ctrl saline vs. cKO saline	-243	-1959 to 1473	No	ns	>0.9999
60	Ctrl saline vs. cKO saline	-986.9	-2818 to 844.1	No	ns	0.6503
70	Ctrl saline vs. cKO saline	-680	-2859 to 1499	No	ns	0.9825
80	Ctrl saline vs. cKO saline	-621.3	-3250 to 2007	No	ns	0.998
90	Ctrl saline vs. cKO saline	-530.6	-2454 to 1393	No	ns	0.9946
-20	Ctrl Amph1 vs. cKO Amph1	562.3	-859.9 to 1985	No	ns	0.9385
-10	Ctrl Amph1 vs. cKO Amph1	-269.2	-2229 to 1691	No	ns	>0.9999
0	Ctrl Amph1 vs. cKO Amph1	-432	-2995 to 2130	No	ns	0.9999
10	Ctrl Amph1 vs. cKO Amph1	-5525	-9444 to -1606	Yes	**	0.0037
20	Ctrl Amph1 vs. cKO Amph1	544.9	-1597 to 2686	No	ns	0.9965
30	Ctrl Amph1 vs. cKO Amph1	2052	796.5 to 3308	Yes	**	0.0019
40	Ctrl Amph1 vs. cKO Amph1	2151	748.1 to 3555	Yes	**	0.0031
50	Ctrl Amph1 vs. cKO Amph1	2126	633.9 to 3617	Yes	**	0.0053
60	Ctrl Amph1 vs. cKO Amph1	1767	355.4 to 3178	Yes	**	0.0085
70	Ctrl Amph1 vs. cKO Amph1	-356.9	-3526 to 2813	No	ns	>0.9999
80	Ctrl Amph1 vs. cKO Amph1	-1170	-4167 to 1827	No	ns	0.9173
90	Ctrl Amph1 vs. cKO Amph1	-1000	-3122 to 1122	No	ns	0.8134
-20	Ctrl Amph2 vs. cKO Amph2	977.6	102.7 to 1852	Yes	*	0.0205
-10	Ctrl Amph2 vs. cKO Amph2	79.03	-1586 to 1744	No	ns	>0.9999
0	Ctrl Amph2 vs. cKO Amph2	-281.4	-2483 to 1920	No	ns	>0.9999
10	Ctrl Amph2 vs. cKO Amph2	685.7	-1504 to 2876	No	ns	0.9881
20	Ctrl Amph2 vs. cKO Amph2	2946	1396 to 4496	Yes	***	0.0006
30	Ctrl Amph2 vs. cKO Amph2	3014	1606 to 4423	Yes	***	0.0002
40	Ctrl Amph2 vs. cKO Amph2	2849	1314 to 4384	Yes	***	0.0008
50	Ctrl Amph2 vs. cKO Amph2	2644	1043 to 4244	Yes	**	0.0018
60	Ctrl Amph2 vs. cKO Amph2	1289	-1438 to 4017	No	ns	0.8256
70	Ctrl Amph2 vs. cKO Amph2	-1382	-5683 to 2919	No	ns	0.9767
80	Ctrl Amph2 vs. cKO Amph2	-2921	-5709 to -131.6	Yes	*	0.0361
90	Ctrl Amph2 vs. cKO Amph2	-2998	-5232 to -764.5	Yes	**	0.0043
-20	Ctrl Amph3 vs. cKO Amph3	791	-484.9 to 2067	No	ns	0.5241
-10	Ctrl Amph3 vs. cKO Amph3	-504.1	-2330 to 1322	No	ns	0.9936
0	Ctrl Amph3 vs. cKO Amph3	-1258	-3468 to 952.5	No	ns	0.6
10	Ctrl Amph3 vs. cKO Amph3	1921	-228.3 to 4071	No	ns	0.1075
20	Ctrl Amph3 vs. cKO Amph3	3522	1758 to 5286	Yes	***	0.0004
30	Ctrl Amph3 vs. cKO Amph3	3440	1313 to 5566	Yes	**	0.0021
40	Ctrl Amph3 vs. cKO Amph3	3325	1453 to 5196	Yes	**	0.0011

50	Ctrl Amph3 vs. cKO Amph3	3131	1267 to 4994	Yes	**	0.0016
60	Ctrl Amph3 vs. cKO Amph3	1965	-438.5 to 4368	No	ns	0.18
70	Ctrl Amph3 vs. cKO Amph3	-60.89	-3281 to 3159	No	ns	>0.9999
80	Ctrl Amph3 vs. cKO Amph3	-3054	-6523 to 416.1	No	ns	0.1132
90	Ctrl Amph3 vs. cKO Amph3	-3650	-6610 to -690.1	Yes	**	0.0096
-20	Ctrl Amph4 vs. cKO Amph4	559.9	-626.7 to 1746	No	ns	0.7795
-10	Ctrl Amph4 vs. cKO Amph4	-1070	-3340 to 1199	No	ns	0.803
0	Ctrl Amph4 vs. cKO Amph4	-2095	-4570 to 379.8	No	ns	0.1389
10	Ctrl Amph4 vs. cKO Amph4	2640	100.3 to 5180	Yes	*	0.0374
20	Ctrl Amph4 vs. cKO Amph4	4252	2366 to 6138	Yes	***	0.0002
30	Ctrl Amph4 vs. cKO Amph4	3990	2273 to 5708	Yes	***	0.0001
40	Ctrl Amph4 vs. cKO Amph4	3682	1996 to 5369	Yes	***	0.0002
50	Ctrl Amph4 vs. cKO Amph4	3378	1402 to 5353	Yes	**	0.0014
60	Ctrl Amph4 vs. cKO Amph4	3051	1452 to 4651	Yes	***	0.0006
70	Ctrl Amph4 vs. cKO Amph4	2036	-165.0 to 4236	No	ns	0.0851
80	Ctrl Amph4 vs. cKO Amph4	-1651	-5702 to 2400	No	ns	0.8958
90	Ctrl Amph4 vs. cKO Amph4	-4597	-7550 to -1645	Yes	***	0.0009
-20	Ctrl Challenge vs. cKO Challenge	-433	-2063 to 1197	No	ns	0.9962
-10	Ctrl Challenge vs. cKO Challenge	-1203	-3303 to 896.5	No	ns	0.6085
0	Ctrl Challenge vs. cKO Challenge	-2256	-4397 to -115.3	Yes	*	0.034
10	Ctrl Challenge vs. cKO Challenge	3423	1251 to 5596	Yes	***	0.001
20	Ctrl Challenge vs. cKO Challenge	5561	3680 to 7442	Yes	****	<0.0001
30	Ctrl Challenge vs. cKO Challenge	5440	3468 to 7412	Yes	****	<0.0001
40	Ctrl Challenge vs. cKO Challenge	5215	3272 to 7159	Yes	****	<0.0001
50	Ctrl Challenge vs. cKO Challenge	5045	3087 to 7003	Yes	****	<0.0001
60	Ctrl Challenge vs. cKO Challenge	4873	2903 to 6843	Yes	****	<0.0001
70	Ctrl Challenge vs. cKO Challenge	4684	2664 to 6704	Yes	***	0.0001
80	Ctrl Challenge vs. cKO Challenge	2884	-480.6 to 6249	No	ns	0.1387
90	Ctrl Challenge vs. cKO Challenge	479.5	-4562 to 5521	No	ns	>0.9999
